# Associations between phloem microbiota and metabolomes in three North American ash species (*Fraxinus* spp.) susceptible to emerald ash borer (*Agrilus planipennis*)

**DOI:** 10.1186/s40793-026-00884-w

**Published:** 2026-04-01

**Authors:** Judith Mogouong Tambue, Claire Yager, Kathryn E. Bushley

**Affiliations:** 1 School Integrative Plant Sciences, College of Agricultural and Life Sciences, Cornell University, Ithaca, NY USA; 2 Emerging Pests and Pathogens Unit, USDA-ARS, Ithaca, NY USA

**Keywords:** Microbiome, Emerald ash borer, Ash, *Fraxinus* spp., Bacteria, Fungi, Widely targeted metabolomics, Insect gut microbes

## Abstract

**Background:**

Microorganisms play crucial roles in the survival and fitness of their plant and insects hosts, including invasive species. The emerald ash borer (*Agrilus planipennis*, Fairmaire; EAB) is an invasive insect from Asia. It represents a significant threat to North American forest ecosystems, causing widespread mortality in susceptible native ash (*Fraxinus*) species. While previous studies have shown differences in specific plant defense metabolites between susceptible North American ash species and their more resistant Asian counterparts, widely targeted metabolite profiles and their interactions with phloem microbiota in response to EAB infestation has thus far received little attention. This study aimed to profile microbial communities associated with ash phloem and EAB larval guts and their relationship to ash phloem metabolites in three native susceptible North American ash species: *F. pennsylvanica* (green ash), *F. nigra* (black ash) and *F. americana* (white ash).

**Results:**

Using metabarcoding to characterize the microbial communities associated with the larval gut and host tree phloem and widely targeted metabolomics to establish the first global metabolomic profile of phloem in these ash species, we examined interspecies differences in profiles and associations of ash phloem microbiota and metabolites in relation to EAB infestation. Multivariate analysis revealed that fungal communities were distinct in all ash species, while *F. pennsylvanica* (green ash) harbored bacterial communities distinct from black ash. Only black ash showed a phloem profile significantly associated with EAB attack symptoms and had the largest number of differentially abundant bacterial taxa. In contrast, larval gut bacterial communities from green ash were distinct from those in other ash species. Green ash displayed a distinct global metabolite profile from the other two species and had the highest number of differentially regulated metabolites, while black ash had the least. Green and white ash shared a strong upregulation of terpenoid compounds, several of which were among compounds significantly associated with microbial communities in green ash phloem or the EAB larval gut.

**Conclusions:**

Our results provide the first comparative analysis of phloem-associated microbial communities and metabolomes across three susceptible North American ash species and their response to EAB. We found that microbiota and metabolites in green ash showed a distinct response to EAB infestation from the other ash species and we identified specific metabolites exhibiting significant correlations with microbial communities in ash phloem or the EAB larval gut. These findings contribute novel insights into interspecies variability in host-associated microbial communities and metabolomes and their response to an invasive insect.

**Supplementary Information:**

The online version contains supplementary material available at 10.1186/s40793-026-00884-w.

## Background

Microorganisms are important players in numerous ecological functions [1], including plant-insect interactions [2–4]. Many are commensals without obvious positive or negative effects on plant health [5], while others are beneficial symbionts promoting plant health [6] through performing nitrogen cycling [7] or contributing to resistance to biotic stresses, such as herbivorous insects [8–11], or plant pathogens [12–15] that pose threats to both managed and natural ecosystems. Although many studies have investigated the leaves or stems of herbaceous plants, only a few have focused on the phloem tissue of woody hosts [16], a unique plant tissue involved in transporting sugars, amino acids, hormones, and other metabolites that may influence plant-biotic interactions [17].

Insect hosts are also colonized by microorganisms throughout their body, including the exoskeleton [18] and associated specialized structures for symbiotic fungi (i.e. mycangia) [19, 20], the gut, fat body and other cell types [20]. The majority of insects rely on symbiotic microbes to break down compounds such as cellulose so they can utilize them as nutrients, which are often essential for the survival [21], nutrition [20, 22], synthesis of essential compounds [20, 23], and overall fitness [24–26] of their hosts. For wood-boring and phloem feeding beetles that lack obvious external organs for carrying symbiotic microbes, the gut microbiome has been proposed to be essential for the degradation of lignocellulose [27, 28] and plant defensive compounds [29, 30]. These functions of symbiotic microorganisms contribute to the adaptative capabilities of their insect host [31, 32] and could enhance their ability to expand their range. For example, Zhang, et al. [33] suggested that the gut microbiome of *Hyphantria cunea* (Lepidoptera) could enable more rapid adaptation, enhancing the insect’s ability to attack new hosts. More recently, Wang, et al. [34] have suggested that the insect microbiome may enhance suppression of plant-induced defenses and enable its geographic spread and invasion of new habitats. Other studies reported changes in the microbiome correlated with the spread and establishment of invasive insects [19, 35], suggesting these microorganisms may assist in the invasion process.

The emerald ash borer (*Agrilus planipennis* Fairmaire; EAB), a member of the Buprestid family native to the Asian continent [36], is an invasive forest pest first detected in North America in 2002 [37]. The EAB is a phloem feeding specialist that feeds on ash (*Fraxinus* spp.) trees, which are ecologically [38, 39], culturally, and economically [39] important in forest ecosystems of North America. EAB larvae chew through ash phloem, and by doing so they impair critical functions in the host plants, including water and mineral acquisition [40]. When signs of relatively high insect density appear (e.g. canopy dieback, epicormic branching, woodpecker damage), the ash tree dies quickly within two to four years [39]. The EAB is considered the most damaging and costly wood boring pest in U.S. history [39, 41] and continues to spread despite attempted control. As of March 2025, it has been detected in 37 US states and six Canadian provinces (https://www.emeraldashborer.info, accessed 21 January 2026).

The invasive spread of EAB may be due in part to lack of effective plant defenses against this exotic invader, or ‘defense free space’ [38]. With their long lifetime, trees can be exposed to a variety of environmental stresses, including insect attacks, and can evolve defense mechanisms to these challenges [42]. Plant defense mechanisms involve both constitutive and induced metabolites. Substances synthesized by plants to defend themselves against insects include production of toxic chemicals [43–45], sticky resins [42, 46], and volatiles [47, 48] that repel insects, recruit beneficial defenders such as parasitoids, or modify insect behavior. Despite the increasing number of studies contributing to a better understanding of the defense mechanisms of *Fraxinus* spp., our knowledge remains incomplete. Of the 20 native ash tree species occurring in North America [49], the most abundant species are *Fraxinus pennsylvanica* (green ash), *F. nigra* (black ash) and *F. americana* (white ash). These are also among the most susceptible species [50–52], despite phylogenetic studies reporting that black ash is closer to the more resistant native Asian Manchurian ash (*F. mandshurica)* [49, 51].

Constitutively expressed compounds may contribute to insect resistance of different ash tree species. Eyles, et al. [53] found that the more resistant Manchurian ash showed higher concentrations of some compounds that were toxic or deterrent to herbivorous insects. For example, pinoresinol (lignan) which is known for its antifeedant activity [54, 55], verbascoside (phenolic acid) that was reported to decrease survival and growth of EAB larvae in susceptible ash species [56], and esculin (coumarin) which showed anti-microbial activity, had higher constitutive levels in resistant ash species [57]. Although some research studies have suggested a chemical signature for the genus *Fraxinus* [53, 58–60], or compared the relative abundance of specific classes of compounds (lignans, phenolics, others) between the resistant native Asian ash species and the susceptible native North American ash species, no study has comprehensively characterized the global metabolite profiles of the three most common susceptible *Fraxinus* species (green, white, and black ash) distributed across the EAB invasive range in North America. In this study, we use a widely targeted metabolomic approach to broadly profile all classes of compounds occurring in the ash phloem from trees both with and without visible signs of EAB infestation (e.g. presence of galleries, epicormic branching, woodpecker damage).

The role of insect and/or plant microbiomes in the invasion process and their interactions with plant defense are less well understood, yet dissecting the interplay between plant defense metabolites and the microbiome in this insect-host system is crucial to inform future management using either chemical or biological control approaches. Wang et al. [61] suggested that an adaptive microbiome of EAB could suppress plant-induced defenses and enable its geographic spread. Among the compounds previously identified in *Fraxinus* spp., coumarins [62] and phenolics [63] were suggested to play an important role in plant defense. These and other phloem metabolites may also shape the tree microbiome in ways that impact plant-insect-microbe interactions. Our recent study comparing the phloem mycobiome between a resistant native Asian ash species (Chinese ash, *F. chinensis*) and a susceptible ash species native to North America (velvet ash, *F. velutina*), for example, suggested that the taxonomic structure of the fungal community was driven by the host tree metabolite profile [16].

Research aiming to disentangle insect-plant-microbe interactions has been growing quickly in the last decade, mainly because of its ecological importance in the face of new pest pressures resulting from globalization. In this study, we used the widely targeted metabolomics approach on phloem samples from both visibly infested (with galleries) and trees lacking visible signs of EAB infestation of three susceptible North American ash species, coupled with characterization of the microbial communities of these phloem samples and those found in the guts of EAB larvae feeding on these trees. We asked: i) Do phloem and larval gut microbiomes differ in alpha and beta diversity among ash species and by EAB infestation status? ;(ii) Are there taxa enriched in phloem and/or galleries of EAB infested trees? ; (iii) Do shifts in metabolite profiles differ across the three ash species in relation to EAB infestation? ; and (iv) Are phloem metabolite profiles associated with particular microbial taxa in the phloem or larval guts?

## Methods

### Tree selection and sample collection

**Fig. 1:**
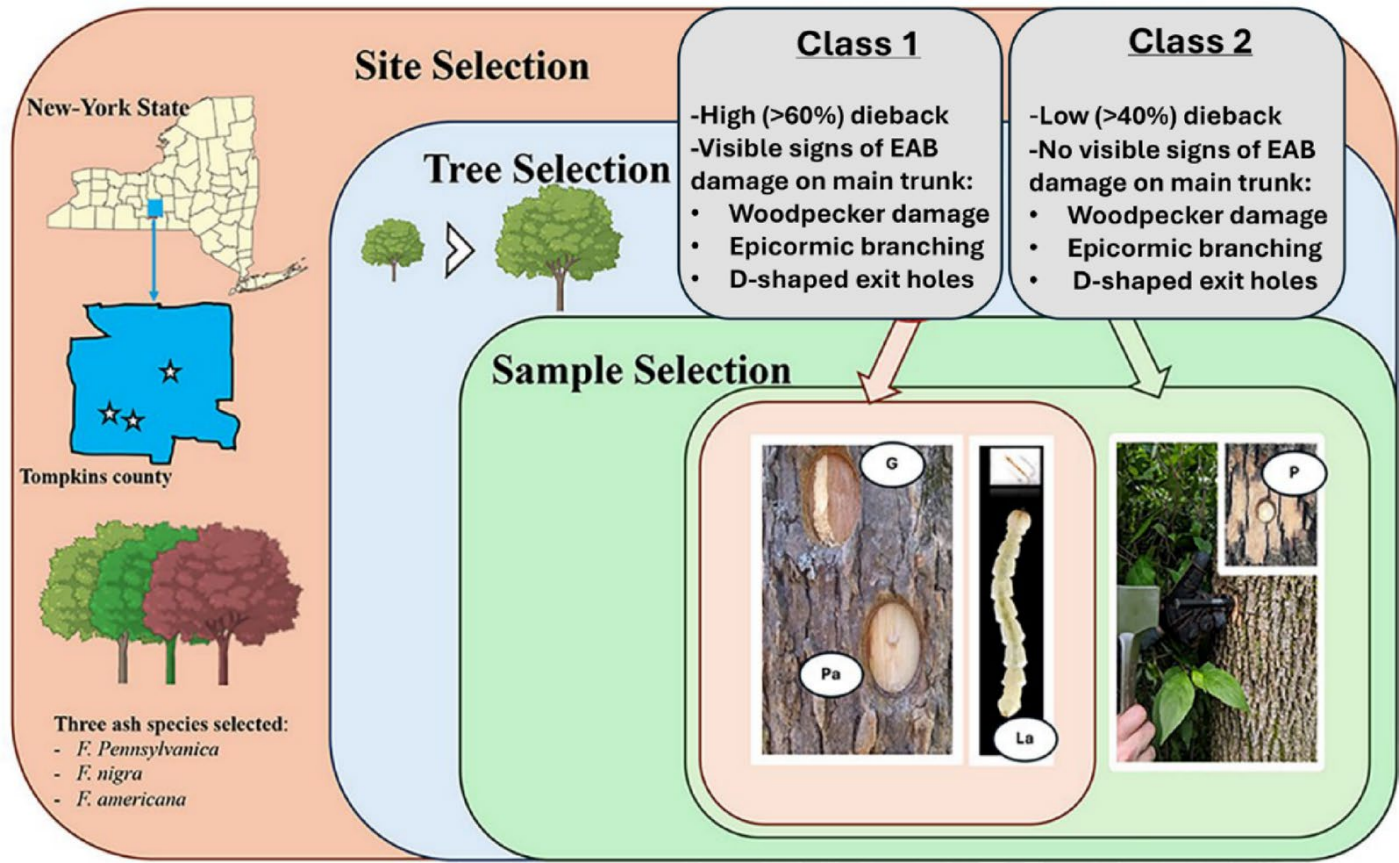
Experimental design and sampling scheme. Trees from three native North American ash species (nine *F. pennsylvanica*, nine *F. nigra* and ten *F. americana* were sampled from Treman State Park and the Renwick woods natural area in Stewart Park, the City of Ithaca, New York. For each tree, each sample type (Phloem – P, PhloemA – Pa and Gallery – G) was represented by two phloem punches collected from two distinct positions around the circumference of the tree at diameter breast height (~ 1 m) and pooled for each replicate tree. For the microbial profiling, three types of sample were collected from ash trees showing visible signs of EAB infestation on the trunk and high dieback (> 60%) (Class 1): (1) uninfested phloem with no visible infestation, but located adjacent to a gallery (PhloemA – Pa), (2) infested phloem from a gallery (Gallery – G), and (3) larvae collected from an S-shaped gallery (Larvae – La). From trees showing no visible signs of infestation on the trunk and low dieback (< 40%) (Class 2), only uninfested phloem with no visible galleries (Phloem – P) was collected

Trees from three native North American ash species, including *F. pennsylvanica* (green), *F. nigra* (black), and *F. americana* (white) were sampled from natural forests in state or city parks near Ithaca, NY, U.S.A. Ten *F. pennsylvanica* trees from Renwick woods natural area in Stewart Park, City of Ithaca (latitude 42.46037898, longitude − 76.50254798, approx. elevation: 122 m), nine *F. nigra* from Robert H. Treman State Park (latitude 42.39894499, longitude, − 76.59105099, approx. elevation: 299 m), and ten *F. americana* trees from Robert H. Treman State park (latitude 42.40109797, longitude − 76.58934301, approx. elevation 316 m) (Fig. 1). For each ash species, we selected an approximately equal number of trees from two categories: Class (1) trees showing visible external signs of EAB infestation such as D-shaped exit holes, epicormic branching, woodpecker damage, and S-shaped larval galleries on the main trunk and high dieback (> 60%), and Class (2) trees lacking visible signs of EAB infestation such as epicormic sprouting, bark flecking by woodpeckers, or evidence of galleries in the main trunk and showing relatively low dieback (< 40%) (Fig. 1, Table S1). All trees in this field study were located in a similar natural environment with potential exposure to EAB as well as other biotic pathogens (e.g. canker, wilt, or root rot pathogens). The sampling design included both a comparison of phloem samples between trees from Class 1 and from Class 2 as well as a paired design sampling two different sample types (uninfested phloem and galleries) from individual Class 1 trees to investigate differences between these sample types while reducing the potential variation between individual trees.

For the microbial community profiling, three types of samples were collected from Class 1 ash trees showing visible signs of EAB infestation: (1) uninfested phloem located adjacent to a gallery (PhloemA – Pa), (2) infested phloem from a gallery (Gallery – G), and (3) larvae collected from an S-shaped gallery (Larva – La) (Fig. 1). From Class 2 trees showing no visible signs of infestation on the trunk, only uninfested phloem (Phloem – P) was collected. All the samples were collected in the late summer to fall season (August 9 to September 30) to sample larvae actively feeding in galleries. For each tree, each phloem sample type (Phloem, PhloemA and Gallery) was represented by two phloem punches collected from two distinct positions around the circumference of the tree at diameter breast height (DBH) using a 0.5-inch phloem punch, stored in labelled sterile bags, and transported to the lab on ice. These two punches were pooled as a single sample per tree to capture potential within tree variation across vertically oriented phloem tissue. The punch was wiped with ethanol and stored in 95% ethanol between samples. Any larvae that were found in an S-shaped gallery were collected using clean insect tweezers, put in 70% ethanol upon collection, and stored at − 20 °C prior to dissection and DNA extraction. Where feasible, additional galleries were sampled from high infestation trees to collect at least five larvae per tree for gut dissections.

For the metabolomics profiling, only the PhloemA and the Gallery sample types, each represented by two pooled punch samples, were collected from Class 1 trees showing signs of EAB infestation to track the ‘within-tree’ metabolite shifts in response to EAB infestation for each ash species while reducing ‘between tree’ variation. All the samples were stored at -80 °C prior to DNA extraction and metabolomics analysis.

## Microbial community profiling

### Sample preparation and DNA extraction

For the phloem and the gallery samples, the bark layer was first gently removed from the punches using sterile gloves and the two phloem punches per tree were pooled to represent a single sample per tree. Therefore, the replication unit was an individual tree. The Phloem, PhloemA, and Gallery samples were each ground separately in an autoclaved mortar and pestle with liquid nitrogen, mixed thoroughly, and 100 mg of tissue powder subsampled for DNA extraction. For each ash tree species, five larvae at stages 3–4 were collected from four Class 1 infested trees (Table S2). To determine the stage of the larvae, the length of their urogomphi were measured under a dissecting microscope (Olympus, SZX9) and only the larvae of instar 3 or 4 were selected based on urogomphi length > 0.43 mm [64, 65]. Each larva was first washed two times in 70% ethanol for 30 s, rinsed in ultrapure water, and then dissected using sterile PBS 1X and autoclaved dissection instruments. After dissection, the five guts were pooled to represent a single sample per tree in order to get sufficient DNA for sequencing, then rinsed with autoclaved ultrapure water, placed in 50 µL of PBS 1X, and ground with an autoclaved micropestle prior to DNA extraction. All the DNA extractions were performed using the Qiagen DNeasy PowerSoil Pro™ kit following a previous study [16] using the manufacturer’s recommendations with the following modifications: The first step of the extraction was done using a bead beater, Bead ruptor Elite Omni Bead Mill Homogenizer, and bead-beaten twice at 6 m/s for 55 s with a 5 min cooling interval on ice. Negative extraction controls that contained no plant or insect tissue were also included.

### PCR amplification, amplicon sequencing, data processing

The v3-v4 region of the 16S rRNA gene for bacteria and the ITS1 rRNA region for fungi were sequenced using the primers (V3_357F_Nextera 5’- CCTACGGGAGGCAGCAG-3’ and V4_806R_Nextera 5’- GGACTACHVGGGT*TCTAAT-3’) for bacteria and the primers (ITS1F_Nextera 5’- CTTGGTCATTTAGAGGAAG*TAA − 3’ and ITS4_Nextera 5’-TCCTCCGCTTATTGATATGC-3’) for fungi. In order to evaluate our sequencing processing pipeline, fungal and bacterial mock communities constructed from our microbial strain bank, including some plant pathogenic, wood decay, and insect associated fungi previously isolated from EAB [66] galleries, were also sequenced (Table S3). For each sample, a minimum of 10 ng of DNA was submitted to the University of Minnesota Genomics Center (UMGC, Minnesota, USA) for amplification, library preparation, and Illumina MiSeq sequencing with 2 × 300 bp paired-end reads using a two-step dual-indexed amplification method [67]. After removing the adapters using Cutadapt v4.0 [68], sequences were processed in R v4.2.0 (https://cran.R-project.org) using the DADA2 pipeline v1.24.0 [69] to identify amplicon sequence variants (ASVs). For 16 S rRNA gene sequences, reads were filtered with the following parameters: truncLen = c(260,240), maxEE = c(2,3), truncQ = 2, and maxN = 0. For ITS sequences, reads were filtered with maxEE = c(5,5), truncQ = 2, maxN = 0, and minLen = 50 to ensure sufficient overlap of paired-end reads [70]. Chimeras were removed using removeBimeraDenovo(method = “consensus”). Reads filtered at each step in the pipeline were tracked to ensure optimal parameters and that no step lost the majority of reads (Table S4). Taxonomic assignment was performed using the RDP v18 [71] database for 16 S and the UNITE v9.0 database [72] for ITS, both formatted for DADA2. Sequences not classifiable to the bacterial or fungal kingdoms at ≥ 80% bootstrap confidence were discarded. ASVs present in extraction and library prep negative controls were removed from all samples (Table S5).

## Metabolite profiling: widely targeted metabolomics

Samples of PhloemA and Gallery were collected from four Class 1 infested ash trees using the same protocol as for DNA metabarcoding above (two punches pooled per sample type) and stored at -80 °C prior to metabolite extraction. Immediately after being removed from the − 80 °C freezer, the samples were chilled in liquid nitrogen for several minutes for transport to the lyophilizer and lyophilized for 72 h using a freeze-dry system (Labconco, Kansas City, MO, USA). The bark was gently removed, and each sample was wrapped in aluminum foil, labelled, placed in a plastic bag with desiccant, and shipped to MetWare Biotechnology Inc., MA, USA for metabolomics analysis. Samples were ground using a ball mill (30 Hz, 1.5 min) (MM 400, Retsh). For extraction, 50 mg of the ground tissue was mixed with 1.2 mL of -20 °C pre-cooled 70% methanol with internal standards described in Chen, et al. [73]. The mixture was then vortexed (30 s every 30 min for a total of six times, centrifuged (12,000 rpm, 3 min, 4 °C), and the supernatant collected and filtered through a 0.22 μm membrane filter. Extractions were run alongside internal standards on an ultra-performance liquid chromatography and tandem mass spectrometry (UPLC-MS/MS) system (Applied Biosystems Qtrap 6500, https://sciex.com/ accessed 03 April 2025). The UPLC-MS/MS was performed as described in Koski, et al. [16]. An internal QC standard was created by mixing a small aliquot from each sample together and injected four times, once for every ten samples, as technical replicates. The data from the QC sample was used to assess the stability of the process by looking at the concordance of the TIC plot and the CV value of the internal standards, which were < 15%. Metabolites that showed > 30% CV amongst the QC samples were filtered out to reduce noise. All the samples were run as a single batch and thus batch correction was not applied, but the data were visualized in a PCA plot as a quality control step to ensure that samples were tightly clustered. Data was normalized to the sample weight as an equal dry weight of each sample was used for all extractions. The area of each chromatographic peak represents the relative abundance of the corresponding compound, and the mass spectrum peak of each metabolite in different samples was corrected based on retention time and peak distribution information to ensure the accuracy of qualitative and quantitative analysis.

## Statistical analysis of microbiome and metabolome

Statistical analyses were performed using the R software package v4.2.0. Alpha-diversity was calculated using the Hills numbers incorporated in the ‘iNext’ v3.0.0 package [74]. The Chao1 index (q = 0) was used to determine the species richness of each assemblage, and alpha diversity was evaluated using the exponent of the Shannon index (q = 1), which weights species in proportion to their frequency, and the inverse of the Simpson index (q = 2), which places more weight on dominant species. The Wilcoxon test was used to compare the amplicon sequence variant (ASVs) diversity across various sample types. Taxonomic profiles were explored using the ‘phyloseq’ v1.42.0 [75] and ‘ggplot2’ v3.5.1 [76] packages, whereas the shared and unique taxa were investigated using the VennDiagram v1.7.3 package [77]. The homogeneity of the data across the sample types was evaluated prior to betadiversity analysis, using the betadisper() function in the vegan v2.6.4 package [78]. Betadiversity across the various sample types was investigated using cmdscale() incorporated in the ‘stats’ v4.2.0 package [79] using Bray-Curtis distances. The contribution of the microbial communities to the differences observed was evaluated based on PERMANOVA using the adonis() function from the vegan package and post-hoc pairwise analyses using the pairwise.adonis() function from the pairwiseAdonis v0.4.1 package [80]. Differential abundance analysis of microbial taxa identified to the lowest taxonomic rank down to the family and genus level was performed with bias correction using the ANCOMBC v2.6.0 package [81] to find potential markers associated with EAB infestation. The ancombc() function was run with the Benjamini-Hochberg correction method, a prevalence cutoff of 0.10, and the structural zeros based on group TRUE. This method was used to compare microbial abundances across all the sample types and identify potential enrichments based on the q-values < 0.05 and a log-fold change > 2.

The metabolomics data were first examined using principal coordinates analysis (PCoA) to examine within and between species differences in global metabolite profiles. Orthogonal partial least squares discriminant analysis (OPLS-DA) was used to identify significantly different metabolites between PhloemA and Gallery sample types and to examine differences in PhloemA profiles between species using the OPLSR.Anal() function in the MetaboAnalystR v4.0 package [82]. The OPLS model evaluation was performed by bootstrapping for 200 times. Following OPLS-DA modeling, the variable importance in projection (VIP) was used in a multivariate analysis to identify differentially abundant metabolites between samples. The criteria for significance were a VIP score > 1 and a *P* value < 0.05 (from the Wilcoxon rank-sum test) for the two-group analysis, whereas only the metabolites with FDR < 0.05 were selected for the multi-group analysis. We used redundancy analysis (RDA) to model the effect of host tree chemistry (1922 metabolites) on its associated phloem microbes and on insect gut associated microbes using a subset of ASVs whose occurrence was observed in at least 20% of the phloem samples (57 fungal ASVs and 250 bacterial ASVs). For the larval gut samples, only the ASVs detected in at least two samples per host tree species were kept (5 fungal AVSs and 28 bacterial ASVs). These cutoffs were used to remove rare ASVs with very sparse distribution across the samples. After selecting variables using the forward selection with the double-stopping criterion [83], the selected predictors were used in a partial RDA after a preliminary Hellinger transformation of the ASV abundance matrices to first test the influence of tree properties, including metabolites. Similarly, after removing rare ASVs for the larval gut samples, the forward selection with the double-stopping criterion allowed detection of predictors for the gut-associated fungi and others for the gut-associated bacteria.

## Results

### Microbial profiling

Across all samples, we obtained a total of 1,616,677 metabarcoding reads for the bacterial (16 S) and 2,255,343 reads for the fungal (ITS) communities. These reads corresponded to 18,396 ASVs for bacteria and 5,402 for fungi.

#### Alpha-diversity

**Fig. 2:**
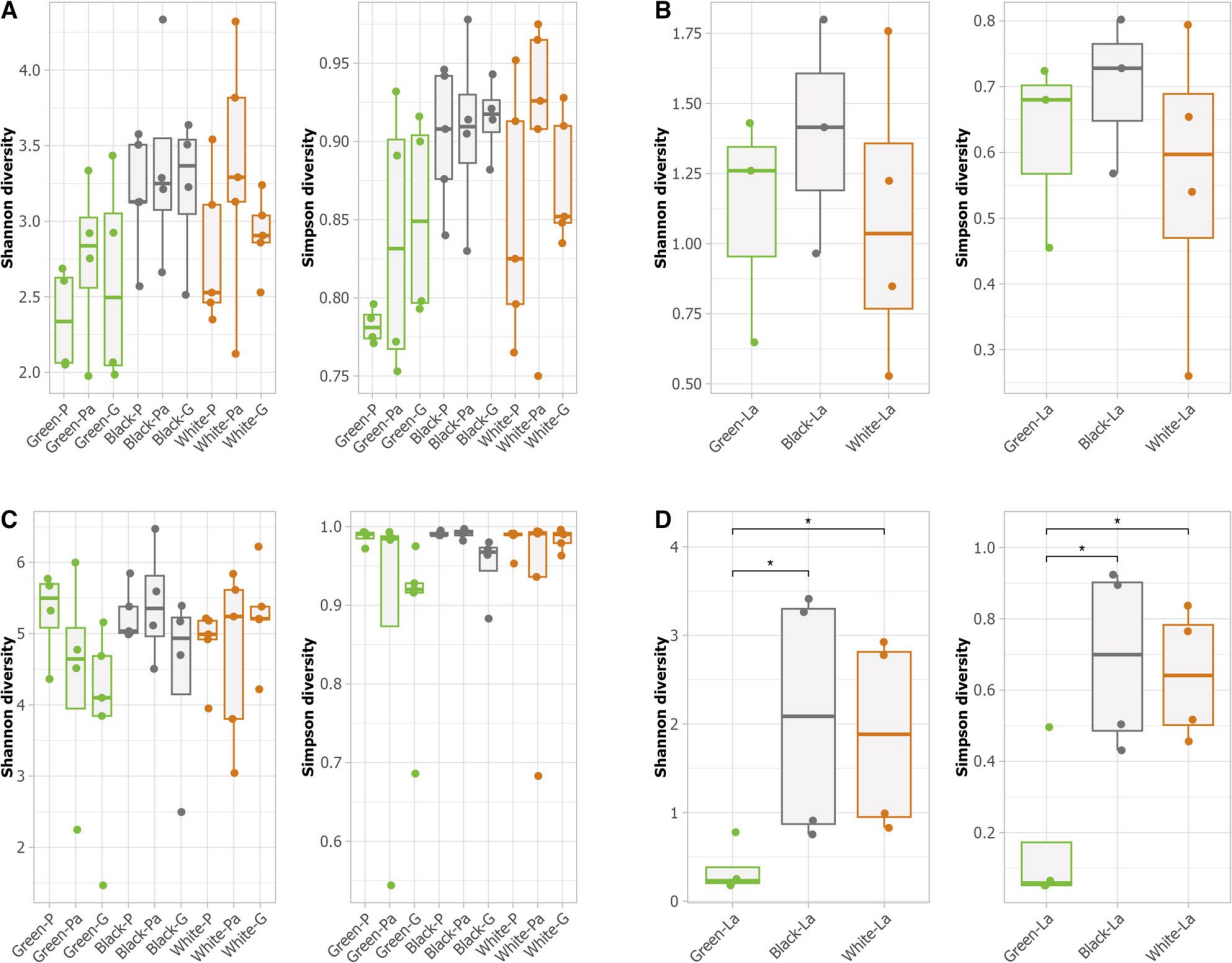
Alpha diversity in the ash phloem and EAB larval gut microbial communities. Fungal community in **A** phloem and **B** larval gut. Bacterial community in **C** phloem and **D** larval gut

Alpha-diversity indices computed to investigate the potential relationships between sample types and ASV diversity did not show any significant differences between the larval gallery (“Gallery” or “G”) and the adjacent phloem samples (“PhloemA” or “Pa”) in Class 1 EAB infested trees, or between the Gallery and the phloem (“Phloem” or “P”) from Class 2 trees based on the Shannon and Simpson indices (*P* > 0.05) (Fig. 2A). Although the bacterial community in phloem also did not show significant differences between any of the phloem sample types, the alpha-diversity in Gallery samples from green ash showed a trend of lower diversity compared to Phloem, as reflected in both the Shannon Green-P (mean: 5.46 ± 0.31) versus Green-G (mean: 4.15 ± 0.59): *p* = 0.024, adj.*p* = 0.18) and Simpson (Green-P (mean: 0.99 ± 0.01) versus Green-G (mean: 0.90 ± 0.04): *p* = 0.04, adj.*p* = 0.19) indices. For black ash, the Simpson index also tended to be lower in the Gallery (mean: 0.95 ± 0.02) compared with both PhloemA (mean: 0.99 ± 0.01), *p* = 0.02, adj.*p* = 0.17 and Phloem (mean: 0.99 ± 0.01), *p* = 0.04, adj.*p* = 0.19) (Fig. 2B, Table S6).The fungal communities did not show this same trend.

Both fungal and bacterial communities associated with the larval gut also showed a trend of lower alpha-diversity compared to communities associated with phloem based on both Shannon (guts: 1.19 ± 0.44 and phloem 2.94 ± 0.60 for fungi; guts: 1.44 ± 1.26 and phloem: 4.89 ± 1.04 for bacteria) and Simpson (guts: 0.62 ± 0.17 and phloem 0.87 ± 0.07 for fungi; guts: 0.50 ± 0.09 and phloem: 0.95 ± 0.09 for bacteria) indices (Table S6). The diversity of the bacterial larval gut community from the green ash was significantly lower than in the other two ash species according to Shannon (Green-La (mean: 0.36 ± 0.14) versus Black-La (mean: 2.09 ± 0.74): *P* = 0.05, Green-La versus White-La (mean: 1.88 ± 0.56): *P* = 0.05) and Simpson (Green-La versus Black-La: *P* = 0.05, Green-La versus White-La: *P* = 0.05) indices (Fig. 2B). In contrast, the fungal larval gut community was not significantly different between ash host species (Fig. 2B, Table S6).

#### Taxonomic composition

**Fig. 3:**
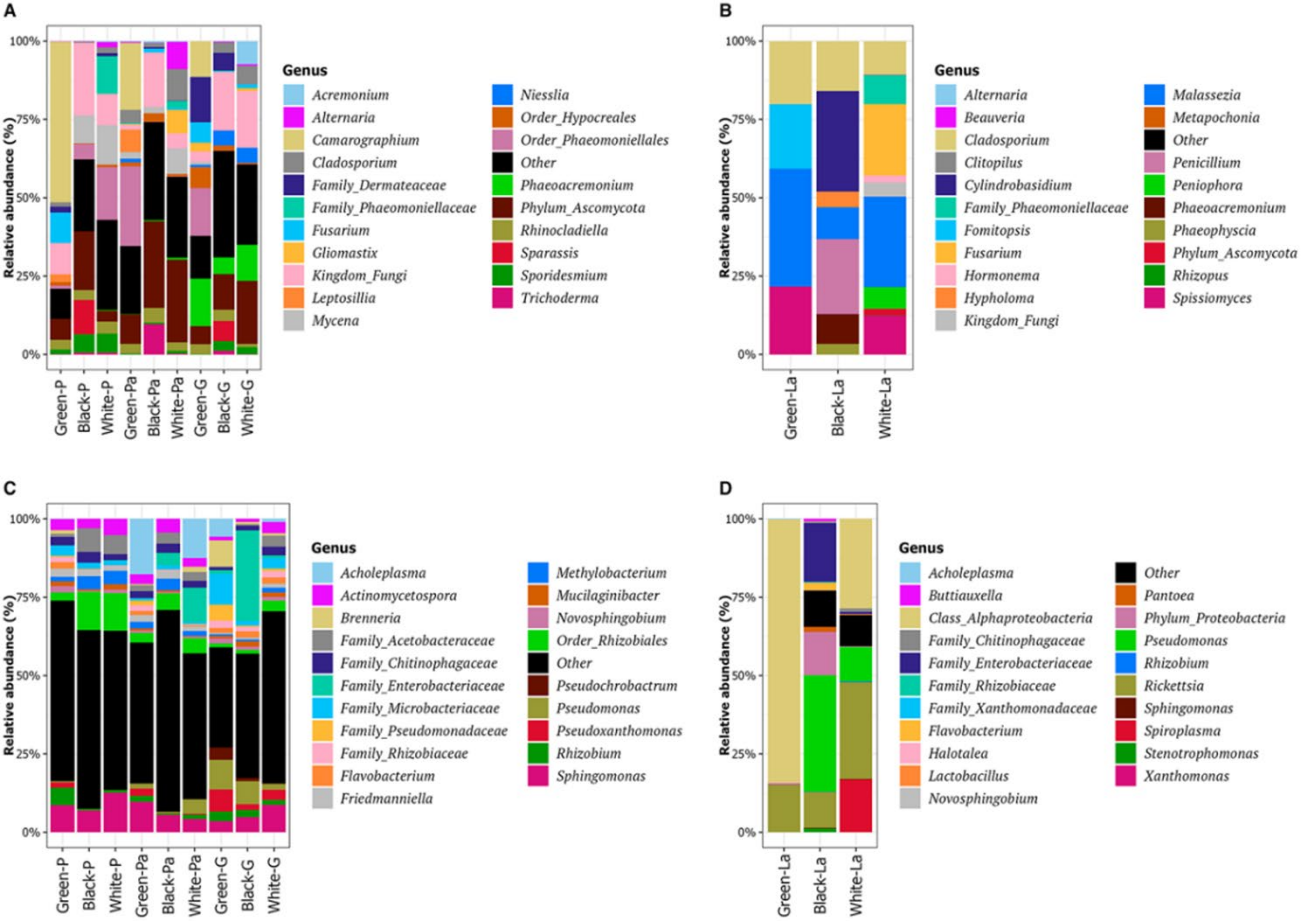
Taxonomic profile of top 20 genera with highest relative abundance in the ash phloem and EAB larval gut microbial communities. Fungal community in (**A**) phloem and (**B**) larval gut. Bacterial community in (**C**) phloem and (**D**) larval gut

The fungal community was represented by six phyla, dominated by the Ascomycota (average of 75% of taxa in the phloem and 46% in the larval gut) and the Basidiomycota (average of 14% of taxa in the phloem and 53% in the larval gut) (Fig. S1). The Basidiomycota in the larval gut belonged predominantly to the classes Agaricomycetes and Malasseziomycetes, whereas Basidiomycota in phloem belonged to a wider range of classes (Agaricomycetes, Exobasidiomycetes, Microbotryomycetes, Tremellomycetes) (Fig. S2). Among the fungal taxonomic classes found in the larval gut, more than 80% of the ASV corresponded to three classes, Agaricomycetes in Basidiomycota and Dothideomycetes and Malasseziomycetes in Ascomycota (Fig. S1, Table S7). This contrasted with the phloem samples, where more than 50% of the ASVs were represented by Ascomycota in Classes Dothideomycetes, Eurotiomycetes and Sordariomycetes. The Sordariomycetes and Leotiomycetes represented a greater proportion of the fungal community in Gallery samples (Fig. S3), with the three orders Hypocreales (Sordariomycetes), Helotiales (Leotiomycetes), and Togniniales (Sordariomycetes) having highest relative abundance (Fig. S1). Among the top twenty genera with highest relative abundance in the fungal community, four (*Cladosporium*,* Rhinocladiella*,* Sporidesmium* and *Phaeoacremonium*) were detected in every sample type of all three ash species. Other taxa were detected only in a single ash species. The genus *Leptosillia*, for example, was found only in the Phloem and PhloemA of green ash, while the genera *Sparassis* and *Lepraria* were found only in the black ash samples, and *Botryosphaeria* was only detected in the white ash Gallery (Table S7). In all three ash species, the genera *Phaeoacremonium* and *Niesslia* showed higher relative abundance in the Gallery, whereas the genus *Mycena* showed higher relative abundance in the Phloem and PhloemA (Fig. 3A). The three genera comprising the greatest proportion of taxa in the fungal community from the insect’s gut varied across host tree species: *Fomistopsis* (41%), *Cladosporium* (30%) and *Malassezia* (21%) in larval guts collected from green ash; *Cylindrobasidium* (49%), *Penicillium* (21%) and *Malassezia* (11%) in larval guts collected from black ash; *Spissiomyces* (39%), *Peniophora* (22%) and *Malassezia* (12%) in larval guts collected from white ash. However, two genera (*Malassezia* and *Cladosporium*) were found in all the larval guts (Fig. S1, Table S7).

The bacterial community was represented by 15 phyla but was dominated by the Proteobacteria, which represented a higher percentage of the insect gut communities (average of 46% in the phloem and 86% in the larval gut). Although the Actinobacteria phylum was present in all the sample types, it comprised a higher percentage of taxa in the phloem and less than 1% of taxa in the larval gut communities (average of 36% in the phloem and 0.19% in the larval gut) (Fig. S1, Table S7). Among the bacterial classes found in the larval gut, the Alphaproteobacteria was dominant in the guts collected from green and white ash, representing 99% and 61% of the total bacterial community, respectively, whereas the Gammaproteobacteria were dominant in the larval gut collected from black ash (58%) (Fig. S4, Table S7). In Gallery samples, the Xanthomonadales and Pseudomonodales tended to have higher relative abundances, with the orders Actinomycetales, Rhizobiales and Enterobacteriales showing highest relative abundance (Fig. S4). The bacterial community shared a larger number of genera cooccurring in the top twenty taxa across all the phloem sample types compared to the fungal community, although with varied relative abundances. The genus *Sphingomonas* had the highest relative abundance in most phloem sample types, representing 3 to 12% of the total taxa. The genera, *Pseudomonas* and *Pseudoxanthomonas*, tended to have higher relative abundance in the Gallery sample types compared to others (*Pseudomonas* (7.7% ±2.8 (G), 2.6+-2.3 (Pa) and 0.2 ± 0.2 (P)) and *Pseudoxanthomonas* (4.8 ± 1.4 (G), 0.7 ± 0.4 (Pa), 0.8 ± 0.7 (P)), whereas the relative abundance of some ASVs assigned to the order Rhizobiales tended to show higher relative abundance in the Phloem compared to the Gallery samples for the white and black ash species (12.0 ± 0.1 (P), (4.4 ± 0.2 (Pa) and 1.7 ± 0.5 (G)) (Figs. 3B, S4). Among the top twenty genera with highest relative abundance in the larval guts, *Pantoea*, *Pedobacter* and *Cellulomonas* were detected only in the larval gut from black ash, *Acholeplasma* and *Halotalea* in the larval gut from green ash, and *Spiroplasma* only in the larval gut from white ash (Fig. 3C). In contrast, some genera were detected in all larval guts across all three ash species, including *Rickettsia*, *Pseudoxanthomonas*, *Clostridium_sensu_stricto*, *Pseudomonas*, *Sphingomonas*, *Actinomycetospora*, *Flavobacterium*, *Friedmanniella*, and *Caulobacter*. Among these shared genera, *Rickettsia* had highest relative abundance, representing 19%, 17% and 57% of taxa for green, black and white ash species, respectively. (Fig. 3D, Table S8).

#### Beta-diversity

**Fig. 4:**
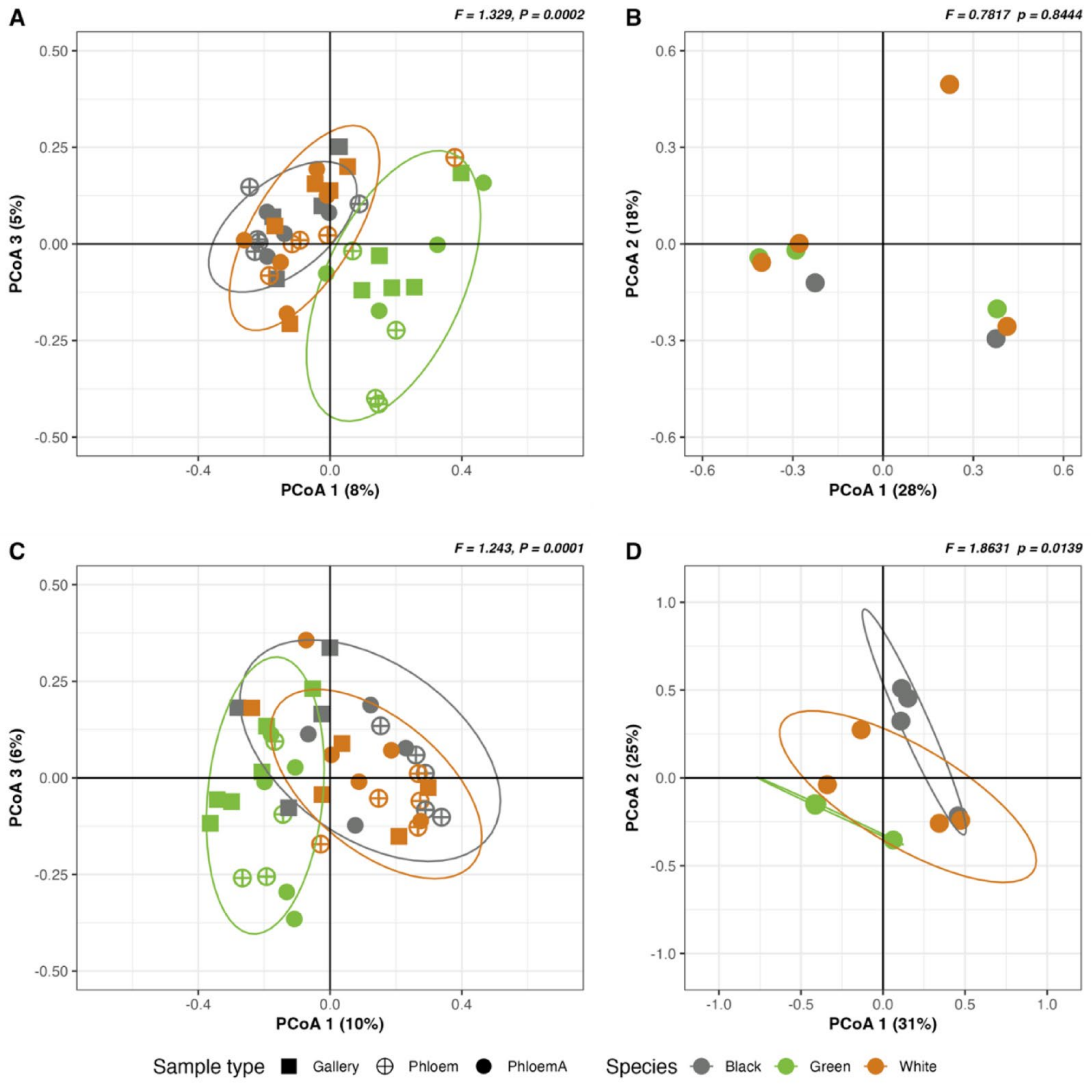
PCoA of beta-diversity of fungal community based on Bray-Curtis distances. Fungal community in (**A**) phloem and (**B**) larva and bacterial community in (**C**) phloem and (**D**) larva. Ash species are color coded (green ash = green, black ash = black, white ash = orange) and phloem sample types are coded by shapes (Gallery = square, Phloem = unfilled circle, PhloemA = solid filled circle). Ellipses indicate 95% confidence intervals

For the phloem samples, the betadispersion of the microbial ASVs did not differ between the species, nor between the sample types (Fungal community: *P* = 0.266 for the distribution across species and *P* = 0.566 for the sample types, bacterial community: *P* = 0.213 for the species and *P* = 0.241 for the sample types) (Table S9). Analyses of beta-diversity across all phloem sample types (Phloem, PhloemA, Gallery) using PCoA based on Bray-Curtis distances revealed significant differences in the fungal community associated with phloem of the three ash species (R^2^ = 0.249, F = 1.329, *P* = 0.0002, Fig. 4A, Table S9). More specifically, the fungal communities of the three ash species differed significantly from each other, with green ash showing more significant differences from the other two ash species (Green-P versus Black-P: F = 1.893, adjusted *P* = 0.0001, Green-P versus White-P: F = 1.693, adjusted *P* = 0.001) than black from white ash (Black-P versus White-P: F = 1.315, adjusted *P* = 0.011) (Table S9). No significant difference was found between the different sample types within each ash species (Table S9). The EAB gut associated fungi showed no significant differences based on the ash host species (*P* = 0.843, Fig. 4B, Table S9).

The bacterial community associated with the phloem also revealed significant differences across the three ash species (R^2^ = 0.243, F = 1.243, *P* = 0.0001, Fig. 4C, Table S9), with green ash being distinct from black ash (Green-P versus Black-P: F = 1.982, adjusted *P* = 0.037, Table S9), whereas no significant differences were observed between green and white or between white and black ash (adjusted *P* > 0.05, Table S9). Unlike the fungal community associated with the larval guts, the bacterial community in larval guts showed significant differences among ash species (F = 1.863, *P* = 0.014, Fig. 4D, Table S9), with bacterial communities in green ash distinct from those associated with the other two ash hosts (Green-La versus Black-La: F = 2.455, adjusted *P* = 0.032; Green-La versus White-La (F = 2.879, adjusted *P* = 0.032, Table S9). Only the black ash showed a significant change in the phloem associated bacterial community in relation to EAB infestation (Black-P versus Black-G: F = 1.999, adjusted *P* = 0.037, Fig. 4C, Table S9).

#### Taxa enriched in EAB galleries compared to phloem in each ash species

The ANCOM-BC analysis performed between sample types in each species revealed some taxa significantly enriched in relation to the EAB infestation status between Class 1 and Class 2, or between sample types (e.g. Phloem and/or PhloemA vs. Gallery). For the fungal community, no fungal markers were found differentially enriched in the white ash at the family level (Table 1), Green ash showed one marker *Neopyrenochaetaceae* increased with a log-fold change (LFC) of 2.14 and 2.34, respectively and one marker at the genus level (*Capronia*) (Table S10) enriched in both PhloemA and Gallery compared to Phloem. An additional family level marker in green ash (Togniniaceae, LFC = 5.24), was enriched in Gallery compared to Phloem (Table 1). At the genus level, *Phaeoacremonium*, a genus within the Togniniaceae, was significantly enriched with a large fold increase (LFC = 4.74) in Gallery compared to Phloem (Table S10). In black ash, there were no markers between Phloem and PhloemA identified at the family level (Table 1). No markers were increased in black ash Gallery compared to PhloemA, but two markers showed an increase in Gallery compared to Phloem, including Aspergillaceae (LFC = 4.17) and one taxon mapping to order Hypocreales fam. Incertae sedis (LFC = 3.11). At the genus level, *Penicillium*, a genus within Aspergillaceae, was increased (LFC = 4.08) between Phloem and Gallery. (Table S10). No fungal markers that were enriched in gallery samples was shared across ash species.

For bacteria, three markers were enriched in Phloem compared to Gallery, including Pseudomonadaceae (LFC = 3.59), Xanthamonadaceae (LFC = 2.35), and Verricumicrobia for green ash species (LFC = 2.35) (Table 1). At the genus level, the genus *Pseudomonas* within Pseudomonadaceae was also significantly enriched (LFC = 3.67) in Gallery compared to Phloem (Table S10). For black ash, family Enterobacteriaceae showed an increase (LFC = 3.43) in PhloemA of the Class 1 trees compared to Phloem in Class 2 trees (Table 1). Genera in the family Pseudomonadaceae also showed an increase in Gallery compared to both Phloem (LFC = 2.74) and PhloemA (LFC = 3.94) in black ash (Table S10). The white ash revealed two bacterial markers enriched in PhloemA of Class 1 trees compared to Phloem in Class 2 trees (Order_Gp6; LFC = 2.27 and Class_Spartobacteria; LFC = 2.29) and one marker, Bradyrhizobiaceae, enriched in Gallery (LFC = 2.75) compared to PhloemA (Table 1). Only members of the family Pseudomonadaceae showed a shared increase in Gallery versus Phloem samples in both green and black ash.

**Table 1:** Differential analysis depicting enrichment between all the sample types in each ash species

	Pairs	Fungal marker	LFC	W	Q	Bacterial marker	LFC	W	q
Green ash	Phloem vs. PhloemA	No marker				No marker			
	PhloemA vs. Gallery	Neopyrenochaetaceae	2.34	3.511	0.039	No marker			
	Phloem vs. Gallery	Neopyrenochaetaceae	2.14	3.788	0.012	Phylum_Verrucomicrobia	2.35	4.427	0.001
		Togniniaceae	5.24	3.245	0.048	Xanthomonadaceae	2.35	3.703	0.012
						Pseudomonadaceae	3.59	3.392	0.027
Black ash	Phloem vs. PhloemA	No marker				Roseiarcaceae	− 2.61	− 4.119	0.002
						Beijerinckiaceae	− 2.36	− 4.166	0.010
						Class_Acidobacteria_Gp1	− 2.95	− 3.652	0.014
						Enterobacteriaceae	3.43	3.398	0.016
	PhloemA vs. Gallery	Class_Sordariomycetes	− 4.74	− 4.234	0.002				
	Phloem vs. Gallery	Aspergillaceae	4.17	4.040	0.006	Class_Betaproteobacteria	− 3.10	− 4.308	0.000
		Hypocreales_fam_Incertae_sedis	3.11	3.592	0.012	Class_Acidobacteria_Gp1	− 3.28	− 3.544	0.005
		Ophiocordycipitaceae	− 3.87	− 3.635	0.012	Cryptosporangiaceae	− 2.97	− 3.456	0.007
		Myriangiaceae	− 2.57	− 3.149	0.046	Order_Blastocatella	− 2.30	− 3.278	0.012
						Beijerinckiaceae	− 3.13	− 2.930	0.032
White ash	Phloem vs. PhloemA	No marker				Order_Gp6	2.27	3.751	0.007
						Class_Spartobacteria	2.29	4.013	0.007
						Geodermatophilaceae	− 2.44	− 3.240	0.037
	PhloemA vs. Gallery	No marker				No marker			
	Phloem vs. Gallery	No marker				Bradyrhizobiaceae	2.75	3.676	0.031

The first column shows the pairs of sample types used for the comparisons in each ash species. The second column corresponds to the differential family marker detected with the ANCOMBC analysis. The third column “LFC” stands for Log fold change in the pair compared. The fourth column indicates W statistic and the fifth column the q value, the adjusted *p* value obtained by applying the Benjamin-Hochberg method to *p* value

### Metabolomics profiling

#### Global metabolite profiles

**Fig. 5:**
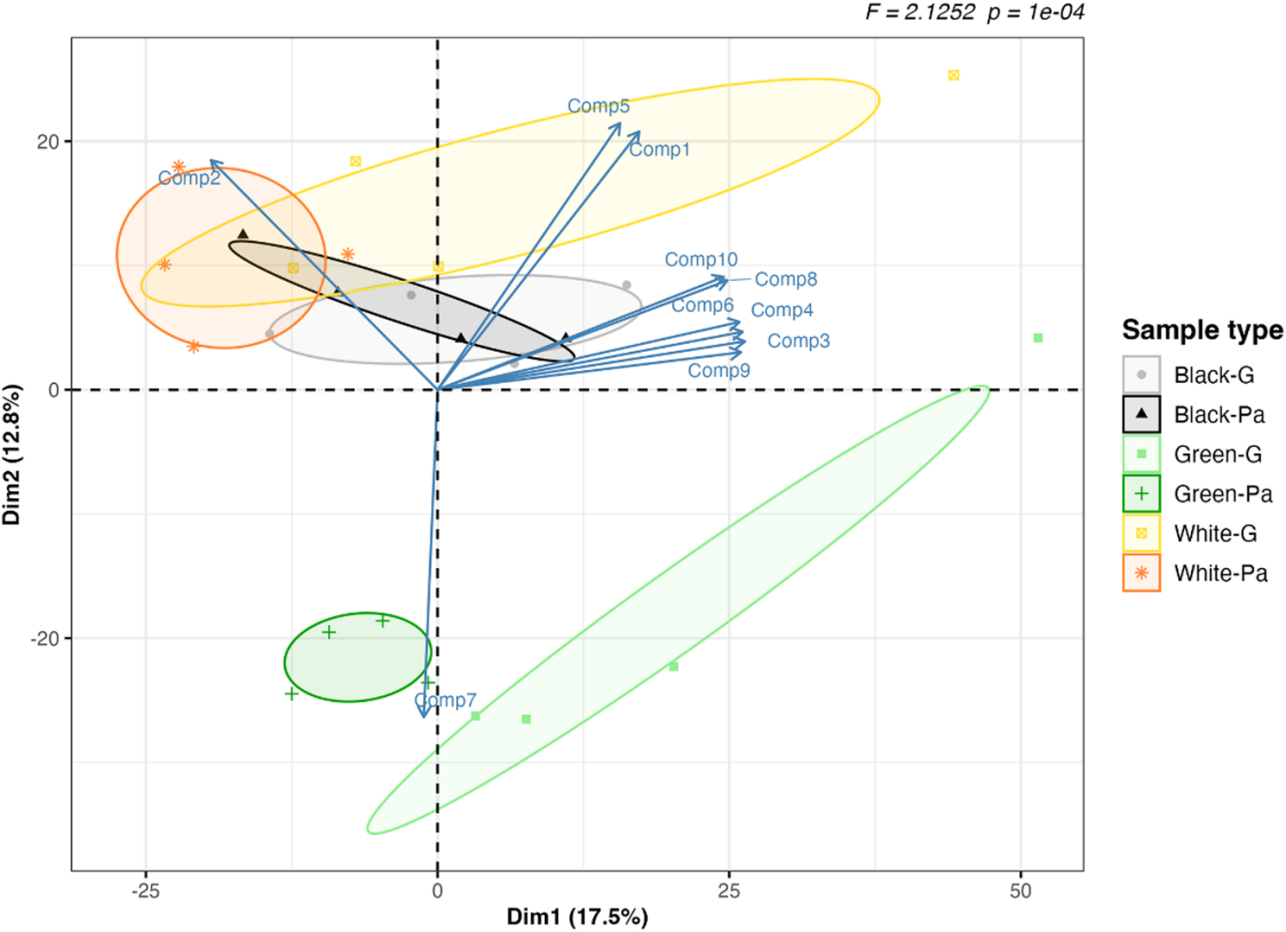
Principal component analysis comparing the within- and among-species differences (PERMANOVA, R^2^ = 0.371, F = 2.125, *P* = 0.0001) of metabolites detected in both PhloemA and Gallery samples. Vectors of the ten compounds (shown in Table 2) contributing most to the model are superimposed. Ash species are color coded (black ash =black, green ash = green, white ash = yellow). Gallery samples are coded as a circular dot for all species, while PhloemA samples are designated with a triangle (black), cross (green), or asterix (white) for each species

The widely targeted metabolomic approach based on UPLC-MS/MS allowed the identification of 1922 metabolites across all 24 phloem and gallery samples. These encompassed eleven compound classes: 17% phenolic acids, 14% lignans and coumarins, 12% lipids, 9% alkaloids, 9% amino acids and derivatives, 7% terpenoids, 3% flavonoids, 3% nucleotides and derivatives, 3% organic acids, 2% quinones, and 19% in a class named ‘others’ including compounds such as alcohols and aldehydes (Fig. S5, Table S11). The principal component analysis (PCA) representation indicated a significant separation of samples based on their metabolites, PERMANOVA R^2^ = 0.371, F = 2.125, *P* = 0.0001; homogeneity of dispersions: p = 0.452 (Fig. 5, Table S12), with post-hoc pairwise comparisons showing green ash as significantly different from the other two ash species (Green-Pa versus Black-Pa: R^2^ = 0.247, F = 1.971, adjusted *P* = 0.046, Green-Pa versus White-Pa: R^2^ = 0.296, F = 2.528, adjusted *P* = 0.046, Table S12). Additionally, only the green ash showed a significant change in metabolite profiles in relation to EAB infestation (Green-Pa versus Green-G: R^2^ = 0.247, F = 1.963, adjusted *P* = 0.046; Fig. 5, Table S12). The ten metabolites contributing most to the PCA model included three saccharides (L-fucitol, D-xylonic acid and D-arabitol), two triterpenes (3α,20β,21β-trihydroxy-16-oxo-serrat-14-en-24-oic acid and 2,3,19-trihydroxy-24-oxo-olean-12-en-28-oic acid), a lignan (5’-methoxyisolariciresinol-9’-O-glucoside), a phenolic acid (sinapoylglucuronic acid), a vitamin (6-galloylglucosyl ascorbic acid), a ketone (3,4’-dihydroxy-3’,5’-dimethoxypropiophenone), and a nucleotide derivative (adenine) (Fig. 5). More of these top ten metabolites were upregulated in Gallery compared to PhloemA in green ash (7) compared to white (3) and black (0) ash (Table 2).

Among the 1922 compounds, three were detected only in the PhloemA in all ash species, including one alkaloid (4-Hydroxyquinoline) and two phenolic acids (ethyl maltol and benzoyltartaric acid) (Fig. S6, Table S11). Similarly, three compounds were detected only in the Gallery, namely γ-L-glutamyl-L-[S-(4-hydroxyphenylbenzyl)]cysteine glycine (amino acid and derivatives), ditartaroyl-hydroxycoumarin (coumarin) and 4-O-glucosyl-sinapate (phenolic acid). Moreover, some compounds were detected only in the PhloemA and not the Gallery and vice versa in green ash (thirteen detected only in Gallery, nine detected only in Phloem A), black ash, (eight detected only in Gallery, eight detected only in PhloemA), and white ash (twelve detected only in Gallery, ten detected only in PhloemA) (Fig. S6, Table S11). Fraxetin glucuronide and 2-propylsuccinic acid were detected only in the PhloemA of black and white ash but not in PhloemA of green ash (Fig. S6, Table S11). A differential analysis comparing the PhloemA of the three ash species using OPLS analysis revealed 15 compounds contributing most to the interspecies differences between the three ash species, including compounds belonging to the terpenoid, lignan, phenolic acid, and lipid classes (Fig. S7, Table S13).

**Table 2:** Ten compounds contributing most to the PCA model of phloem metabolites and their relation to EAB infestation

Contribution rank	Index	Class II	Compounds	Formula	Regulation detected (PhloemA to Gallery)		
					Green ash	Black ash	White ash
Comp1	Sacch16	Saccharides	L-Fucitol	C6H14O5	Up	–	up
Comp2	Lign14	Lignans	5’-Methoxyisolariciresinol-9’-O-glucoside	C27H36O12	Down	–	–
Comp3	Terpen95	Triterpene	3α,20β,21β-trihydroxy-16-oxo-serrat-14-en-24-oic acid	C30H46O6	–	–	–
Comp4	PhenolA116	Phenolic acids	Sinapoylglucuronic acid	C17H20O11	Up	–	–
Comp5	Sacch33	Saccharides	D-Xylonic acid	C5H10O6	Up	–	up
Comp6	Terpen53	Triterpene	2,3,19-Trihydroxy-24-oxo-olean-12-en-28-oic acid	C30H46O6	Up	–	up
Comp7	Nucleo65	Nucleotides derivatives	Adenine	C5H5N5	–	–	–
Comp8	Vita17	Vitamin	6-Galloylglucosyl ascorbic acid	C19H22O15	Up	–	–
Comp9	Sacch61	Saccharides	D-Arabitol	C5H12O5	Up	–	–
Comp10	Keto11	Ketone compounds	3,4’-Dihydroxy-3’,5’-dimethoxypropiophenone	C11H14O5	Up	–	–

Significant up or down regulation from PhloemA to Gallery samples (VIP score > 1 and P-value < 0.05) is indicated for each ash tree species in the three last columns

#### Changes in metabolomic profiles in EAB galleries

**Fig. 6:**
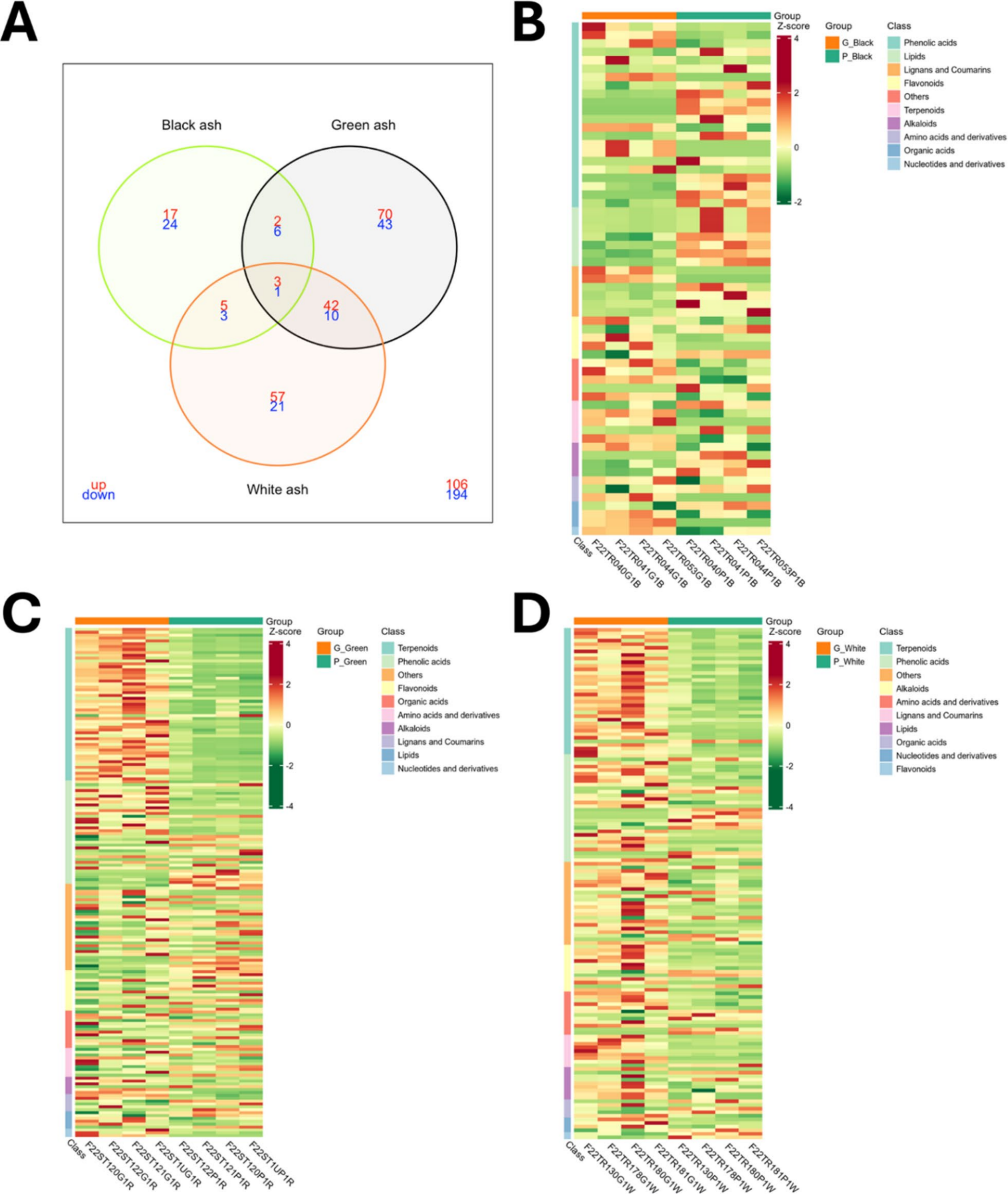
OPLS analysis between PhloemA and Gallery samples within each species revealed distinct patterns of significantly up- or down-regulated metabolites in response to EAB infestation. (**A**) Venn diagram of up-regulated (red) and downregulated (green) metabolites across ash species. Heatmaps of metabolite relative content for differentially regulated metabolites in (B) black ash, (**C**) green ash, and (**D**) white ash

The OPLS analyses revealed 303 compounds significantly up- or down-regulated in galleries infested with EAB larvae (green ash: R^2^X = 0.541, Q^2^ = 0.644, black ash: R^2^X = 0.490, Q^2^ = 0.45, white ash: R^2^X = 0.470, Q^2^ = 0.580, Figs. S8, S10). Overall, green and white ash showed a greater metabolic response to EAB infestation, with a larger total number of differentially regulated compounds in green (177) and white (142) ash compared to black ash (61) (Fig. 6A). Among the 303 differentially abundant compounds, 52 were shared between green and white ash (42 upregulated and 10 downregulated), whereas only eight were shared between black and green ash (two upregulated and six downregulated), and eight between black and white ash (five upregulated and three down-regulated) (Fig. 6C-D). Green and white ash had a greater proportion of upregulated versus down-regulated metabolites, while black ash showed more down-regulated metabolites (Fig. 6A, Table S14). For green and white ash, the terpenoid class had the highest number of compounds differentially regulated in response to EAB infestation (e.g. PhloemA versus Gallery sample types), while the greatest number of compounds significantly differentially regulated in black ash belonged to the phenolic acids class (Fig. 6C-E). Four compounds showed a similar pattern of regulation across all three ash species, with two phenolic acids (alpha-asarone and 4-O-glucosyl-sinapate) and a triterpene (19-hydroxy-3-oxo-24-norolean-12-en-28-oic acid) upregulated in all species, while a quinoline alkaloid (4-hydroxyquinoline) was downregulated (Fig. 6A, Table S14). Interestingly, eight of the ten compounds contributing most to the PCA model were also found significantly differentially regulated in green ash, with one, the 5’-methoxyisolariciresinol-9’-O-glucoside (lignan) decreasing and seven other compounds increasing from the PhloemA to the Gallery (Fig. 5; Table 2). Similarly, ten of the fifteen compounds found contributing most to the interspecies differences between the three ash species in the OPLS analysis comparing the PhloemA of the three species also showed significant changes, mostly upregulation in response to EAB infestation (Table S13).

### Relationship between phloem phytochemistry and microbiota in different ash species

**Fig. 7:**
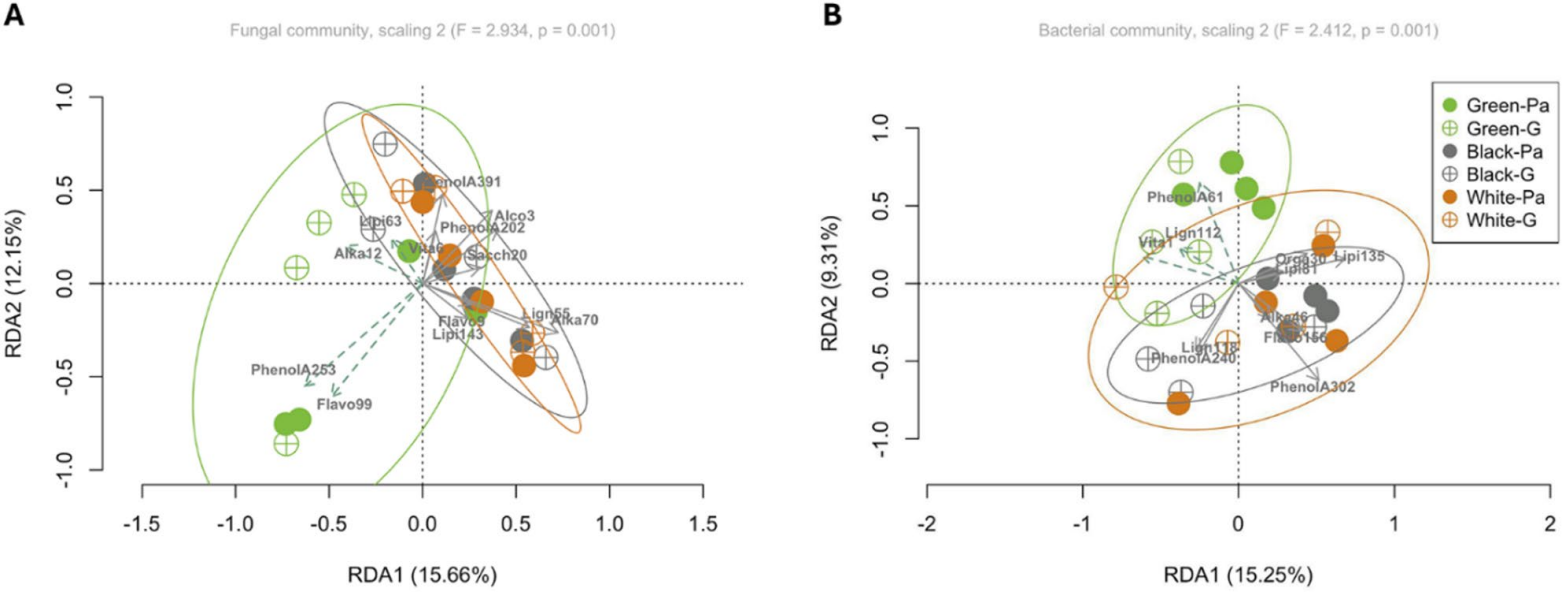
Representation of scaling 2 of the RDA showing the relationships between the PhloemA metabolites and microbial communities in phloem (PhloemA and Gallery sample types). (A) Fungal community. The two first axes were significant (axis 1: F = 7.538, *p* = 0.001; axis 2: F = 5.850, *p* = 0.001) and together explain 28% of the variation and display the thirteen compounds showing significant associations with the fungal community (adjusted R^2^: 0.522). The green dashed arrows show the compounds positively associated with the fungal communities from green ash (*F. pennsylvanica*) phloem, including the two strongest drivers, isorinic acid (PhenolA263; phenolic acid) and chrysoeriol-6,8-di-C-glucoside-4’-O-glucoside (Flavo099; flavone). (B) Bacterial community. The two first axes were significant (axis 1: F = 5.875, *p* = 0.001; axis 2: F = 3.586, *p* = 0.001) and together explain 25% of the variation and display the eleven compounds showing significant associations with the bacterial community (adjusted R^2^: 0.403). The green dashed arrows show the compounds positively associated with the bacterial communities in green ash (*F. pennsylvanica*) phloem, namely orotic acid (Vita1; vitamin), matairesinoside (Lign112; lignan) and isomartynoside (PhenolA61; phenolic acid). The strongest driver, 3,6’-disinapoylsucrose (PhenolA302; phenolic acid), showed a negative association with the bacterial community in green ash (Table S15). The *p* values and coordinates for significant compounds and axis in the RDAs are shown in Table S16, respectively

From the 1922 metabolites found in the phloem samples, the forward selection allowed selection of several predictors sharing significant relationships with the phloem microbial communities represented by 57 fungal ASVs and 250 bacterial ASVs. Thus, 13 compounds explained over half (adjusted R^2^: 0.522) of the taxonomic structure of the phloem fungal community, including four compounds all positioned in the same quadrants as the coordinates of the green ash in the RDA analysis and positively associated with green ash: isorinic acid (PhenolA253; phenolic acid), O-acetyl-L-carnitine (Alka12; alkaloid), dodecanoic acid (Lipi63; free fatty acid), and chrysoeriol-6,8-di-C-glucoside-4’-O-glucoside (Flavo99; flavone) (Fig. 7A; upper and lower left quadrants, Tables S15 and S16). Although no compounds were found significantly positively or negatively associated with Gallery versus Phloem samples, four of these 13 compounds showed significant differential regulation associated with EAB infestation between PhloemA and Gallery in OPLS analysis, including upregulation of O-acetyl-L-carnitine (Alka12; alkaloid) and syringaresinol-4’-O-(6’’-acetyl)glucoside (Lign55; lignan) in green ash, and upregulation of isorinic acid (PhenolA253; phenolic acid) and downregulation of a lipid compound (Lipi153; 1-(2,3-Dihydroxypropoxy)-3-(((2-(dimethylamino)ethoxy)(hydroxy) phosphoryl)oxy) propan-2-yl (8E,11Z,14Z)-octadeca-8,11,14-trienoate) in white ash (Fig. 6A, Tables S13 and S14).

The community composition of the bacterial community was driven by 11 compounds that explained around 40% of their taxonomic structure (R^2^: 0.403), including three compounds with a positive association with green ash based on their RDA coordinates on the two first axes (upper left quadrant), namely isomartynoside (PhenolA61; phenolic acid), matairesinoside (Lign112; lignan), and orotic acid (Vita1; vitamin) (Fig. 7A, Tables S15 and S16). As for the phloem fungal community, no clear pattern was found related to EAB infestation (Fig. 7B, Tables S15 and S16). However, two of the 11 compounds showed significant differential regulation in OPLS analysis only in green ash, including down-regulation of the lipid lysoPC 15:0(2n isomer) (Lipi135) and upregulation of the lignans matairesinoside (Lign112) and secoisolariciresinol-9’-O-glucoside (Lign118) (Fig. 7B, Table S14).

Lastly, multiple regression performed using the RDA approach revealed six compounds found in the Gallery that shared significant relationships with the fungal community in the larval gut represented by 5 ASVs. These included a lignan (magnolin), a flavonol (syringetin-3-O-(6’’-acetyl)glucoside), an alkaloid (hexadecyl ethanolamine), a free fatty acid (petroselinic acid), and a nucleotide derivative (nicotinate adenine dinucleotide phosphate) (Table 3). Similarly, six compounds, including three terpenoids (a monoterpenoid, a sesquiterpenoid and a triterpene), two phenolic acids (usnic acid and magnoloside B) and an organic acid (fumaric acid) showed significant relationships with the bacterial community associated with the larval gut represented by 28 ASVs (Table 3).

**Table 3:** Compounds detected in the RDA model of Gallery metabolites as drivers of the taxonomy of the gut microbial structure (fungi and bacteria)

Index	Class	Compound name	VIF	ANOVA	
				F	*p* value
*Compounds in Gallery interacting with larval gut fungal community (n = 20 larvae)*					
Others58	Others	(2E)-3-(1-Hydroxy-2,6,6-trimethyl-4-oxo-2-cyclohexen-1-yl)-2-propenoic acid	2.114	24.804	***
Lign66	Lignans	Magnolin	1.956	24.737	***
Alka16 *Green ash (upregulated)*	Alkaloids	Hexadecyl ethanolamine	1.628	10.963	***
Flavo132 *Black ash (downregulated)*	Flavonols	Syringetin-3-O-(6’’-acetyl)glucoside	1.673	7.250	**
Lipi24	Free fatty acids	Petroselinic acid	1.394	5.578	***
Nucleo10	Nucleotides and derivatives	Nicotinate adenine dinucleotide phosphate	1.222	4.346	*
*Compounds in Gallery interacting with larval gut bacterial community (n = 20 larvae)*					
Monoterp30	Monoterpenoids	Foliasalacioside B1	2.283	9.381	***
Orga24	Organic acids	Fumaric acid	1.247	2.941	*
PhenolA134	Phenolic acids	Usnic acid	1.326	7.034	***
PhenolA390	Phenolic acids	Magnoloside B	1.637	15.111	***
Terpen14	Sesquiterpenoids	(6R,9R)-Megastigman-4-en-9-ol-3-one O-β-D-(6’-O-β-D-apiofuranosyl)glucopyranoside	2.555	2.697	*
Terpen47 *White ash (upregulated)*	Triterpene	2,3,19,23-Tetrahydroxyurs-12-ene-28-oic acid (myrianthic acid)	1.355	8.884	***

Symbol * represents statistically significant differences (*** = *p* value < 0.001, ** = *p* value < 0.01., * = *p* value < 0.05)

## Discussion

This study aimed to provide the first comparative analysis of phloem metabolites, microbial communities, and their associations in infested and uninfested ash phloem and the guts of EAB larvae feeding on this phloem across three susceptible ash species native to the invasive range of EAB in North America. We found relatively diverse microbial communities in ash phloem, which tended to have higher species diversity than those in the larval guts for both fungi and bacteria. Although fungal and bacterial alpha diversity in the phloem samples was similar across the sample types and species (Shannon and Simpson indexes), the bacterial community, but not the fungal community, showed a trend of lower diversity in Gallery samples (Shannon and Simpson indexes). Overall, the fungal community in phloem was distinct in each species, whereas the bacterial community only showed a significant difference between the green and the black ash (Fig. 4, Table S9). Only the black ash showed significant differences between the phloem tissue infested by the larvae (Gallery) and the phloem tissue not infested by the larvae (Phloem). The bacterial community in the larval guts of green ash was distinct from those of larvae collected from the two other ash species. The global metabolite profile of green ash differed significantly from the other ash species and green ash (Fig. 5, Table S11) and had the highest number of differentially regulated metabolites (Fig. 6A, Table S14). The three ash species also showed distinct patterns of differential regulation of metabolite classes between the PhloemA samples and Gallery samples, with green and white ash strongly upregulating terpenoids and black ash strongly upregulating phenolics (Fig. 6). RDA analysis aimed at linking differences in the microbial communities with specific metabolites identified several key compounds from PhloemA samples positively associated with phloem fungal (isoronic acid (phenolic) and chrysoeriol-6,8-di-C-glucoside-4’-O-glucoside (flavone)) and bacterial (orotic acid (vitamin), matairesinoside (lignan) and isomartynoside (phenolic acid)) communities in green ash (Fig. 7). Similarly, several compounds from Gallery samples were associated with the fungal larval gut community including a lignan (Magnolin), a fatty acid (Petroselinic acid), and an alkaloid (Hexadecyl ethanolamine), while three terpenoids, two phenolics, and an organic acid were associated with the larval gut bacterial communities (Table 3).

## Microbial species within ash phloem in relation to EAB infestation

Despite the increasing numbers of studies on microbial communities associated with leaves and other aboveground parts (e.g. stem, floral nectaries) and their roles in combatting environmental and biotic stress in herbaceous plants, the microbial communities associated with phloem tissues in woody plants and their potential roles in plant-insect-microbe interactions are not well documented. To address this knowledge gap, we profiled the bacterial and fungal communities associated with phloem of the three most common susceptible ash species native to North America and the potential changes in these communities in relation to EAB infestation. Fungi play key ecological roles in nutrient acquisition and carbon sequestration in forest ecosystems [84]. Here, the fungal community in the ash phloem was dominated by the phylum Ascomycota, with Eurotiomycetes, Dothideomycetes, and Sordariomycetes as the most abundant classes (Figs. S1, 3) and the orders Hypocreales (Sordariomycetes), Helotiales (Leotiomycetes), and Togniniales (Sordariomycetes) having the highest relative abundance in Gallery samples (Fig. S1). The order Hypocreales includes entomopathogenic fungi that function in controlling insect populations [85–88], while ecological roles in wood decay, and as saprophytes and mycoparasites, have been reported for the Helotiales [89]. The Togniniales, including the genus *Phaeoacremonium* that we found enriched in green ash galleries (Table S10), have been reported as pathogens of various woody hosts [90], including *Vitis vinifera*, *F. latifolia* [91], *F. pennsylvanica* [66] and *Olea europea* [92]. Our results align with a previous study conducted by Held, et al. [66] using a culture-dependent approach to isolate fungi from EAB galleries, including potential canker pathogens, wood decay fungi, and entomopathogens. We detected many of the same potential canker pathogenic genera (*Phaeoacremonium*, *Paraconiothyrium*, *Coniothyrium*, *Thyronectria*, and *Botryosphaeria*), wood decay fungi (*Sistotrema*, *Irpex*, *Peniophora*, *Phlebia*, and *Ganoderma*), and entomopathogenic fungi (*Beauveria*, *Clonostachys*, *Lecanicillium*, *Akanthomyces*, *Microcera*, *Tolypocladium*, and *Metapochonia*) (Fig. 3, Table S7) as this culture-based study. Some fungal isolates from these genera isolated from EAB galleries have been functionally tested and shown to cause cankers (*Phaeoacremonium*, *Thyronectria*) when applied to ash seedlings [93], to degrade lignocellulose in wood chip degradation assays (*Phlebia*) [94], and to infect and kill EAB eggs (*Akanthomyces*, *Lecanicillium*) [95]. Additionally, some of these genera, including entomopathogens such as *Beauveria*, are also endophytes of a wide range of plant species, including some woody plants such as coffee, and are hypothesized to provide protection to plants against insect pests [96, 97]. Further functional studies are needed to assess the potential ecological roles of the fungi we have identified associated with ash phloem and EAB galleries in EAB infestation, plant defense, production of anti-insect metabolites, and biocontrol.

In contrast to the phloem communities, the fungal communities in the larval gut samples were dominated by the phylum Basidiomycota. However, the Basidiomycota taxa found in the larval guts were represented by only two classes (Agaricomycetes and Malasseziomycetes), while those in the phloem were more diverse (Fig. S3, Table S7). Many basidiomycetes colonize dead wood and are able to decompose recalcitrant compounds like lignin and cellulose, contributing to forest carbon cycling [98, 99]. Rajala, et al. [100] reported that Basidiomycota were highly abundant in spruce logs in early to intermediate stages of decay, while Ascomycota were more often associated with advanced stages of wood decay [101], supporting specific ecological functions for these fungi during the process of wood decay. Previous studies have identified the presence of ascomycete fungi (e.g. *Fusarium*) in the gut of wood feeding insects such as the longhorned beetle involved in degrading lignocellulose [102]. We did not detect any fungi with clear roles in lignocellulose degradation in the larval gut, although one of the most abundant genera in the larval gut from black ash, *Cylindrobasidium* (49%) in Agaricomycetes, contains white rot fungi such as *C. torrendii* [103]. More research is needed to investigate the possible role of these fungi in loss of wood integrity in EAB infested trees. Agaricomycetes, the most common class of Basidiomycetes detected, had high relative abundance in both the phloem samples (at least 80% of Basidiomycetes) and in the larval gut (at least 60% of Basidiomycetes), suggesting potential associations with the host insect (Fig. S2). Some studies have reported that multiple Coleoptera species are attracted to fruiting bodies produced by these fungi and use them as food [104]. Among fungal genera in the larval guts, the ascomycete *Cladosporium* was detected in all sample types, while the basidiomycete *Malassezia* had consistently higher relative abundance in larval gut samples compared to the phloem (Fig. 3B, Table S7). While *Malassezia* are best known as human dermatophytes, a number of studies have detected them in insects, including insect guts [105, 106].

One of the early signs of EAB infestation is dieback of the canopy caused by galleries disrupting phloem transport when larvae feed under the bark. Infestation of a tree by EAB starts in the upper canopy where it can begin to cause low levels of dieback, and then works its way down the tree to the trunk in more highly infested trees causing increasing levels of dieback and eventually death as trees lose their capacity to acquire essential nutrients [39]. For our study, infestation status was classified based on both the presence (Class 1) or absence (Class 2) of external infestation signs in the main trunk and dieback ratings [107]. As all trees in this field study had potential exposure to EAB as well as other biotic pathogens (e.g. canker, wilt, or root rot pathogens), the low dieback in Class 2 trees without visible signs of EAB on the trunk could have been due to an early stage of EAB infestation and dieback in all trees sampled could have been impacted by other biotic pathogens. The process leading to the death of trees may also drive changes in the fungal community from healthy phloem tissues to unhealthy or dying tissues. Overall, the species diversity of fungi associated with phloem did not change significantly with EAB infestation status for either fungi or bacteria, but the bacterial community was less diverse in the larval gut collected from the green ash species than those collected from the two other species (Fig. 2, Table S6). The community composition or beta-diversity showed a distinct pattern for the fungal community in each ash species (Fig. 4, Table S9). Our results did not show changes in the fungal community associated with the Gallery samples compared to the other phloem sample types in any of the ash species. It is important to note that other environmental variables could also contribute to the changes observed, as the samples were collected from individual trees in natural ecosystems. Moreover, the results showed a low R^2^ value (R^2^ < 0.24, Table S9). Other variables not included in the model such as microclimate variations, the age of the tree, or the soil composition could have contributed to variation in the microbial communities. Further analyses are required to investigate the potential contribution of the fungal community to the defense mechanism of all three ash species. On the other hand, the bacterial composition in the Phloem samples from green ash was found to be distinct from the community found in the Phloem samples in black ash (R^2^ = 0.22). Contrary to the fungal community, a significant difference was found between the bacterial composition of the Phloem samples from Class 2 trees without visible signs of EAB infestation and Gallery samples from Class 1 trees with signs of EAB infestation in black ash (Fig. 4, Table S9).

Among the fungal marker taxa found enriched in the green ash gallery samples, several have been identified in the literature as plant pathogenic, wood decay, or insect associated fungi: *Capronia*, an ascomycete genus reported as associated with bark beetles and occurring on wood/bark and decaying parts of herbaceous plants and capable to entering the gut of larvae with feeding material [108, 109] and *Phaeoacremonium*, a plant pathogenic canker causing fungi previously reported in EAB galleries in green ash [66] (Table S10). For black ash, Hypocreales_fam_Incertae_sedis was enriched in Gallery compared to Phloem samples (Table 1), while the order Hypocreales was enriched in PhloemA of Class 1 EAB infested trees compared to Phloem of Class 2 trees (Table S10). As noted above, Hypocreales have been reported as plant endophytes and pathogens of insects and several genera of entomopathogenic fungi from Hypocreales have been previously reported from the EAB larval gallery of green ash [110].

The bacterial phloem communities were dominated by Proteobacteria, followed by Actinobacteria. The 20 taxa with highest relative abundance were shared across all phloem sample types (Fig. 3C), likely contributing to the similarity in species diversity observed for bacterial communities across ash species (Fig. 2C). Different studies have reported both increases and decreases in microbial species diversity in plants in response to pathogen invasion [111–114]. We observed a non-significant trend of decreased species diversity between the phloem infested with a larval Gallery compared to the Phloem tissue without EAB infestation in black and green ash trees. Members of the family Pseudomonadaceae were found enriched in the larval Gallery in both green and black ash (Table 1, S10). Among these, the genus *Pseudomonas* was found enriched nearly four-fold in green ash galleries (Table S10). A previous study reported that *Pseudomonas* spp. may benefit bark beetles by providing them with nutrients and protecting them from tree chemical defense [115]. We hypothesize that *Pseudomonas* and other genera in Pseudomonadaceae may play a role in EAB infestation or plant defense against EAB. Future functional studies should investigate the role of *Pseudomonas* and other Pseudomonadaceae in impacting the growth and survival of EAB larvae. The Enterobacteriales was also identified as an enriched marker taxa for the Gallery in black ash (Table 1, S10). Overall, a larger number of bacterial taxa were also identified as enriched or depleted in black ash phloem (Table 1, S10). Combined with the observation that only black ash showed a significant difference in beta-diversity between Gallery and Phloem samples (Fig. 4, Table S9), our results suggest that EAB infestation in black ash species is associated with changes in the composition of the bacterial community in the phloem.

Three bacterial genera (*Sphingomonas*, *Pseudomonas*, *Pseudoxanthomonas*) that have been extensively reported as bacteria associated with coleopteran guts [20] were shared among the 20 taxa with highest relative abundance for both phloem and larval guts samples (Fig. 3C, D). The bacterial communities in the larval gut shared fewer taxa across species than those in phloem, with *Rickettsia*, *Pseudoxanthomonas*, *Clostridium_sensu_stricto*, *Pseudomonas*, *Flavobacterium*, *Actinomycetospora* and *Sphingomonas* showing highest relative abundance. A previous study identified *Rickettsia* as a symbiont in the EAB larval gut [116] and we found that *Rickettsia* had the highest relative abundance of bacterial genera found in the larval gut across all ash species, supporting its role as a gut symbiont of EAB (Fig. 3D). Noticeable differences were observed across larvae from different ash species, with the Alphaproteobacteria more dominant in the larval gut from green and white ash, and the Gammaproteobacteria, including Family Enterobacteriaceae, more dominant in the larval gut from black ash (Fig. S4). The bacterial community in the larval gut from green ash displayed lower alpha-diversity than both black and white ash (Fig. 2), and analyses of beta-diversity found that the larval gut community in green ash was distinct from that in black and white ash (Fig. 4, Table S9).

### Variation in microbial community composition across ash tree species in relation to phylogeny

Host phylogeny and associated genetic traits can influence plant-associated fungal and bacterial communities [117, 118]. Qian, et al. [119] suggested that the species of fungi associated with plants was driven primarily by differences in host plants genetics. Many researchers consider host phylogeny as one of the strongest deterministic drivers of associated microbiomes [120], while others posit that various stochastic drivers, including environmental filters related to the host, such as their metabolites, may play key roles [121]. Of the three *Fraxinus* spp. included in our study, black ash has been reported to belong to a phylogenetic branch in the Bumelioides section, more closely related to the resistant Asian Manchurian ash (*F. mandshurica*), and distantly related to green and white ash which belong to the Melioides Sects. [49, 122]. Our results align with the black ash species having a distinct pattern of both microbiome shifts in relation to EAB as it was the only species showing a significant difference between the Gallery and Phloem samples based on the EAB infestation signs (Fig. 4, Table S9). The ANCOM-BC analysis also found a larger number of microbial marker taxa, particularly for bacteria, for black ash (Table S10). Combined, our data generally support that the phylogeny of the host species may drive a distinct response of the bacterial community in the phloem of black ash species to EAB infestation. Further studies should be conducted to investigate the roles of bacteria in the defense mechanism of the black ash, particularly in relation with wood feeding insects.

### Comparative metabolomic profiling in relation to EAB infestation

The three ash species in this study, *F. pennsylvanica*, *F. nigra* and *F. americana*, are among the most widely distributed and ecologically important native tree species in Eastern North American forest ecosystems [38]. They have also all been reported as being highly susceptible to attack by EAB [50–52], despite phylogenetic studies reporting that black ash and more resistant Asian Manchurian ash (*F. mandshurica*) are closely related [49, 51]. Global comparative metabolite profiling using the widely targeted metabolomic method resulted in the detection of 1922 compounds, encompassing eleven chemical classes with phenolic acids, lignans, coumarins, and lipids having the highest relative abundance. Hydroxycoumarin derivatives were detected in all sample types, aligning with previous studies highlighting these compounds as the characteristic chemical signature of *Fraxinus* species [53, 58–60]. It is noteworthy that the alkaloid 4-hydroxyquinoline, as well as two phenolic acids (ethyl maltol and benzoyltartaric acid), were detected only in PhloemA and not Gallery samples across all species (Table S11). Some hydroxyquinoline derivatives have been successfully tested as botanical insecticides against coleopteran insects [123], exhibiting antifeedant and deterrent activity against several insect pests [124]. Similarly, maltol derivatives produced by conifers [125, 126] also show antifungal [126] and anti-insect activities, for example inhibiting development of spruce budworm larvae [127]. Future studies should test their effects on EAB larvae. Based on the absence or decrease of these compounds in Gallery samples, we hypothesize that EAB larvae may have the ability to degrade these compounds or harbor microbial symbionts able to do so. Conversely, 4-O-glucosyl-sinapate and ditartaroyl-hydroxycoumarin were shared only in the Gallery samples (Table S11). Sinapate derivatives are produced through the phenylpropanoid pathway [128] and are known to be important for regulating lignin biosynthetic enzymes in plants and represent a general response in plant tissues under biotic stress from insect attack [129] or fungal pathogen colonization [130], while hydroxycoumarins have been associated with insect anti-feedant activity [53]. Thus, we hypothesize that detection of these compounds only in the Gallery samples could be a defense mechanism of the tree for starving and killing EAB larvae. Further functional assays exposing larvae directly to these compounds are required to confirm or refute these hypotheses.

All three ash species in this study have been reported to be susceptible to EAB infestation compared to native Asian ash species such as *F. mandshurica* [131] and *F. chinensis* [49, 51]. Nevertheless, the overall PCA analysis showed that green ash metabolite profiles differed significantly from white and black species, which were closely clustered (Fig. 5, Table S12). Additionally, significant differences between metabolite profiles of the PhloemA and the Gallery samples were observed only in green ash in the PCA (Fig. 5, Table S12). Green ash also showed the highest number of both total and uniquely differentially regulated metabolites in response to EAB in OPLS analysis (Fig. 6A), with white ash showing the next highest, and black ash showing substantially fewer. However, overall profiles of metabolite classes differentially regulated in response to EAB showed more similarities between green and white ash, with a strong shared upregulation of terpenoids in both species in Gallery samples (Fig. 6C, D), whereas the largest class of differentially regulated metabolites in black ash belonged to phenolic acids. Thus, while green and white ash shared some similarities in induced responses to EAB that aligns with ash phylogenetic relationships, the PCA analysis suggested that overall, chemical defense mechanism(s) of green ash against EAB may still be distinct from those of the other two ash species (Fig. 5; Table 2). We hypothesize that the lower number of differentially regulated metabolites in black ash could be due to a stronger shift in the microbiome in EAB infested Gallery versus uninfested Phloem tissue discussed above (Fig. 4; Table 1, S10). In particular, we hypothesize that some bacteria enriched in black ash galleries could impact larval survival and thus contribute to plant defense against EAB. Further functional studies are needed to test these hypotheses.

Several compounds previously found in *Fraxinus* spp. in relation to EAB resistance were also detected in our study. Eyles, et al. [53] detected syringaresinol in green ash, as well as verbascoside, syringin and oleuropein hexoside in both green and white ash, while esculetin, fraxetin, and calceolarioside B were detected only in the resistant Manchurian ash, leading them to hypothesize the latter compounds may play a role in resistance to EAB. Our results detected syringin, verbascoside, oleuropein and calceolarioside B in all three ash species, with no significant inter-species variation in relative abundance (Fig. S9, Tables S11, S14). Although we did not detect compounds such as pinoresinol that have also been implicated in resistance to EAB, a derivative was detected (Pinoresinol-4-O-(6’’-acetyl)glucoside) (Table S11). Finally, we detected Vladinol D (lignan) in our previous study comparing native resistant Chinese ash (*F. chinensis*) with the North American susceptible Velvet ash (*F. velutina*) affected by EAB [16]. Although the compounds mentioned above have been proposed to be involved in the ash resistance mechanism [53, 56, 132], none showed constitutive differences in relative abundance in Phloem A among species and only two fraxetin compounds, fraxetin glucuronide and isofraxetin, showed significantly increased relative abundance in Gallery samples in black and green ash, respectively (Table S14) in this study.

Our study using global metabolite profiling instead demonstrated that the terpenoids class of compounds was most strongly upregulated in response to EAB infestation in green and white ash (Fig. 5, Table S14). Among the top 10 metabolites contributing to the PCA model were two triterpenes, one of which was upregulated only in green ash in response to EAB (Table 2). Additionally, among the top 15 compounds contributing most to interspecies differences in OPLS comparing the PhloemA among the three ash species were three terpenoids, two of which were upregulated in white ash and one in both green and white ash (Fig. S9, Table S13). Terpenes are well-known as allelochemicals, important for plant defense and chemical communication [133]. The triterpene norarjunolic acid was among the 15 compounds contributing to inter-species differences in PhloemA profiles (Table S13) and also showed significant upregulation in Gallery samples in green and white ash (Fig. S10, Table S14). Bhakuni, et al. [134] tested and confirmed the growth inhibitory effects of norarjunolic acid on *Spilarctia obliqua* larvae. Our results suggest that future studies should utilize GC-MS and other approaches to investigate the potential effect of norarjunolic acid, as well as other terpenoids, on the response to EAB attack and their effect on EAB larval survival within phloem.

### Associations between the microbial community and metabolites in ash phloem

Tree chemistry contributes to defense against pathogen attack and may shape the plant’s microbiome. This study aimed to evaluate the potential associations between the profiles of the phloem metabolites and microbiota to ascertain the extent to which the microbial community in each ash species was influenced by host tree chemistry. We identified several compounds positively or negatively associated with ash phloem microbial communities (Fig. 7, Table S15). The two most significant drivers positively associated with green ash phloem included isorinic acid (phenolic acid) and chrysoeriol-6,8-di-C-glucoside-4’-O-glucoside (flavone) for phloem fungal and isomartynoside (phenolic acid), matairesinoside (lignan), and vitamin B13 for phloem bacterial communities. Although we did not find a specific association with EAB infestation, some of the compounds strongly associated with the phloem microbial community were also significantly differentially regulated between PhloemA and the Gallery in one or more ash species. Two of the top 13 compounds associated with phloem fungal communities (O-acetyl-L-carnitine (alkaloid) and syringaresinol-4’-O-(6’’-acetyl) glucoside (lignan), were upregulated in green ash in response to EAB. Interestingly, another flavone chrysoeriol derivative (chrysoeriol-7‐O‐glucoside) was found to be a strong driver of the fungal community associated with phloem of an Asian (*F. chinensis)* and a North American (*F. velutina)* ash species, and was downregulated only in susceptible *F. velutina* [16] in response to EAB. Jang, et al. [135] demonstrated the antifungal activity of chrysoeriol 7 extracted from rice against the fungal plant pathogens, *Fusarium graminearum* and *Pythium graminicola*, and other studies have reported antibacterial activity [136, 137]. These results suggest that chrysoeriol derivatives should be further investigated for their role in shaping the ash phloem microbiome and in ash defense responses against EAB and other plant pathogens.

### Associations between tree phytochemistry and the larval gut microbial communities

Previous work characterizing the adult and larval EAB gut in relation to their environment [16, 121, 138] suggested strong interactions between the gut-associated microbes and plant host properties such as tree chemistry. A previous study reported a lignan that increased in response to EAB attack in *F. velutina* and altered the gut bacterial microbiome of EAB larvae feeding on this host [139]. Among the six compounds positively associated with the EAB larval gut fungal community was a lignan (magnolin) that was not differentially regulated in response to EAB (Table 3). We also identified one lignan (Syringaresinol-4’-O-(6’’-acetyl) glucoside) significantly associated with the fungal phloem community and two lignans (secoisolariciresinol-9’-O-glucoside and matairesinol-4’-O-glucoside) significantly associated with the phloem bacterial community that were all upregulated in Gallery samples in green ash (Fig. 5; Table 2).

Of the six compounds significantly interacting with the EAB larval gut bacterial community were three terpenoids: a triterpene upregulated in white ash and a monoterpene and a sesquiterpenoid that were not differentially regulated (Table 3). Among these compounds, the sesquiterpenoids are known to have antifeedant, antibacterial, and antifungal activities and to function as plant growth regulators and antioxidants [140]. Mahmoud and Croteau [141] characterized the monoterpenes as basic weapons of the plant defense against biotic attack, with antiherbivory, antifungal, and antibacterial activities reported [142]. Given the strong up-regulation of many terpenoids in green, and to a lesser extent white ash, an association with the bacterial communities in the larval gut with terpenoids is intriguing and should be further investigated.

### Challenges and limitations

Many previous studies investigating ash defense responses to EAB have used seedling in greenhouse or common garden studies. While this approach represents a more controlled environment and experimental design, these conditions are also somewhat artificial and may not fully capture how EAB impacts the microbiome or metabolomic defense responses of ash trees in natural forest ecosystems. Our field study which aimed to address these responses under natural conditions also comes with some challenges and limitations. Likely because different ash species prefer distinct environmental conditions, we were unable to find sites where all species were represented and thus species was confounded with site in our study design. Although we chose sites in relatively close proximity (< 10 Km) to one another, site-specific factors such as soil, moisture, elevation, microclimate, among others could also contribute to differences we observed in the microbial community or plant metabolome. The low values of the R^2^ obtained in multivariate analysis of some community comparisons in our study illustrate that other factors mentioned above should be considered in future studies. Additionally, gallery samples are only found in highly infested trees, inherently confounding sample type with infestation level. We attempted to control for this by also comparing uninfested phloem (PhloemA) and galleries (Gallery) within Class 1 trees with visible signs of EAB infestation. The highly sparse nature of compositional microbiome datasets, combined with a relatively small sample size, also poses challenges for both multivariate analyses and measures of differential abundance between samples, which should be interpreted with caution. Additionally, multivariate approaches such as RDA can only suggest correlations and associations between taxa and metabolites, not causation. Thus, our study represents a first step in identifying patterns and associations between microbes and metabolites and identifies several hypotheses for further testing using functional bioassays to confirm or refute a role of these microbes in either aiding EAB in infestation or defending trees against EAB.

## Conclusions

This study provides the first global metabolomic profiles of three susceptible native North American ash trees *F. Pennsylvanica* (green ash), *F. nigra* (black ash) and *F. americana* (white ash) and their relationships to fungal and bacterial communities in ash phloem and the guts of EAB larvae feeding on these ash hosts. Our results showed that green ash has a distinct microbial community and metabolomic profile, as well as a larger number of metabolites differentially regulated in response to EAB infestation, compared to the two other ash species. However, black ash was the only species to show a significant difference in microbial communities, particularly bacteria, between EAB infested Gallery and uninfested Phloem samples. We found that terpenoids were the class of metabolites most strongly upregulated in green, and to a lesser extent white ash, in response to EAB infestation, while black ash showed the lowest number of differentially regulated metabolites and upregulated metabolites primarily in the Phenolics class. Additionally, a relationship was detected between several terpenoids and the bacterial community associated with the EAB larval gut. We hypothesize that these compounds could impact the gut bacterial microbiome and potentially the health and survival of larvae feeding on green ash. Lastly, both this study and our previous study identified chrysoeriol derivatives as compounds shaping the structure of fungal communities in ash phloem. This study contributes fundamental knowledge of the associations between ash defense metabolites, and the bacterial and fungal communities in both the ash phloem and the guts of EAB larvae feeding on these hosts, opening up new approaches to manage invasive insect pests such as EAB.

## Supplementary Information

Below is the link to the electronic supplementary material.

Supplementary Material 1.

Supplementary Material 2.

## Data Availability

The metabarcoding dataset supporting the conclusions of this article are available at the Sequence Read Archive of NCBI under BioProject PRJNA1280105 and SRA accessions SRX29272184-SRX29272234 and SRX29552186-SRX29552241. The metabolomics data used in study is available at the NIH Common Fund’s National Metabolomics Data Repository (NMDR) website, the Metabolomics Workbench, https://www.metabolomicsworkbench.org under Study ID ST004301. The data can be accessed directly via its Project DOI: http://dx.doi.org/10.21228/M8FV8K. This site is supported by NIH grant U2C-DK119886. Analyses of metabolomics data supporting the conclusions of this article are also included within the article in additional files 1 (Supplementary Tables) and additional files 2 (Supplemental Figures). All analysis codes and parameters were deposited in the following site: https://github.com/jud-mog/Comparative_profiling_EAB_in_three_native_ash_tree_species.

## Abbreviations

DBH: Diameter at breast height
EAB: Emerald ash borer
OPLS: Orthogonal partial least squares
PCoA: Principal coordinates analysis
PCA: Principal component analysis
VIP: Variable importance in projection
UPLC-MS/MS: Ultra–performance liquid chromatography and tandem mass spectrometry
