# Supplementary material for: Associations between phloem microbiota and metabolomes in three North American ash species (Fraxinus spp.) susceptible to emerald ash borer (Agrilus planipennis)
Source: Environ Microbiome. 2026 Apr 1;21:66. doi: 10.1186/s40793-026-00884-w (PMC13169598; doi:10.1186/s40793-026-00884-w)

Supporting Information

Interactions between phloem microbiota and metabolomes in three North American ash species (*Fraxinus* spp.) susceptible to Emerald Ash Borer (*Agrilus planipennis*)

**Judith Mogouong^1^, Claire Yager^1,2^, Kathryn Bushley^1,2^**

^1^ College of Agricultural and Life Sciences, Cornell University, Ithaca, NY, U.S.A.

^2^ USDA ARSEF, Ithaca, NY, US

jtm297@cornell.edu

keb45@cornell.edu

**Fig. S1.** Taxonomic profile of Phyla and Class across all the sample types (fungal community)


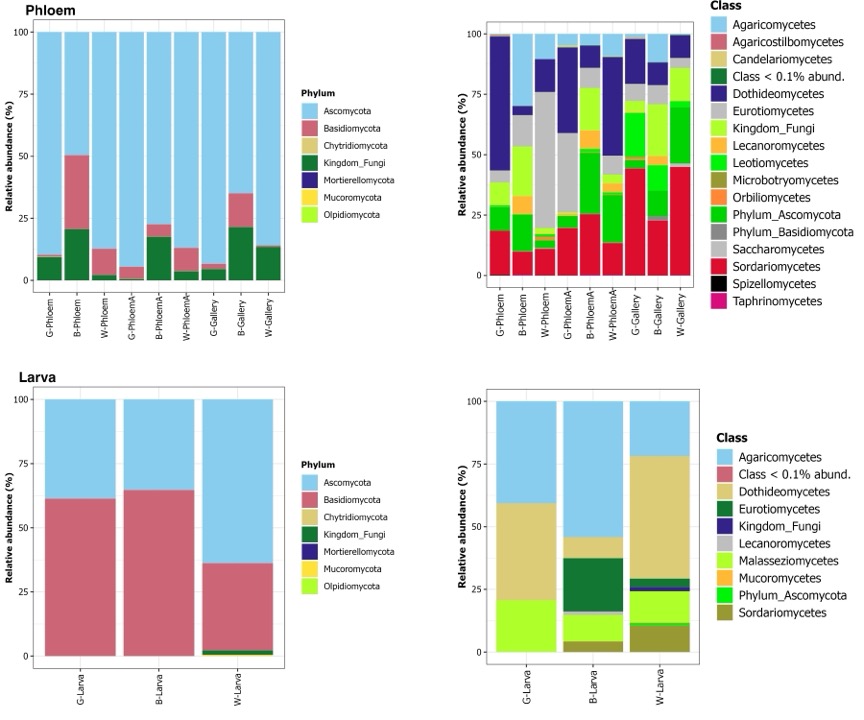


**
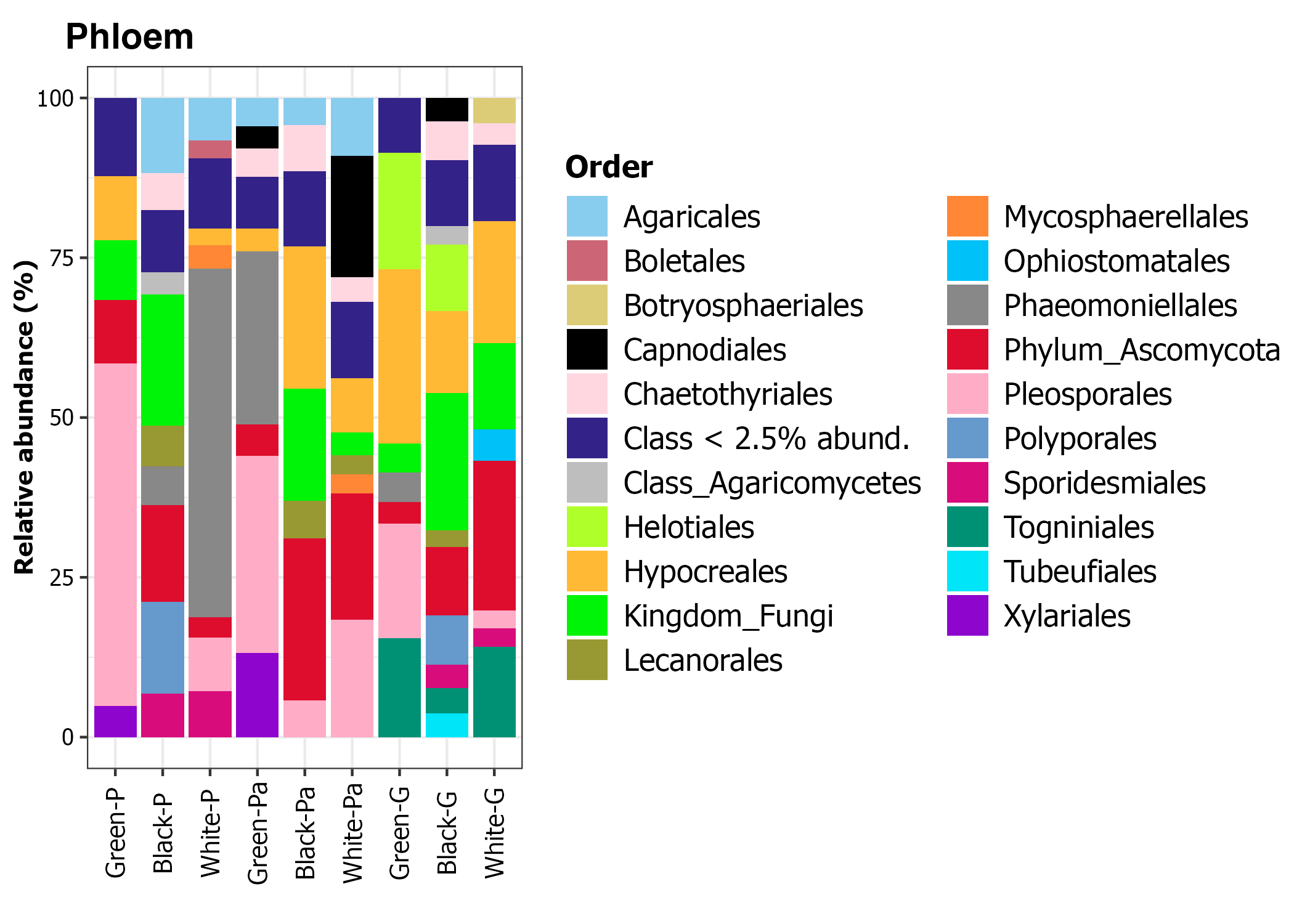
**

**Fig. S2.** Distribution of the Basidiomycetes Classes across all the sample types


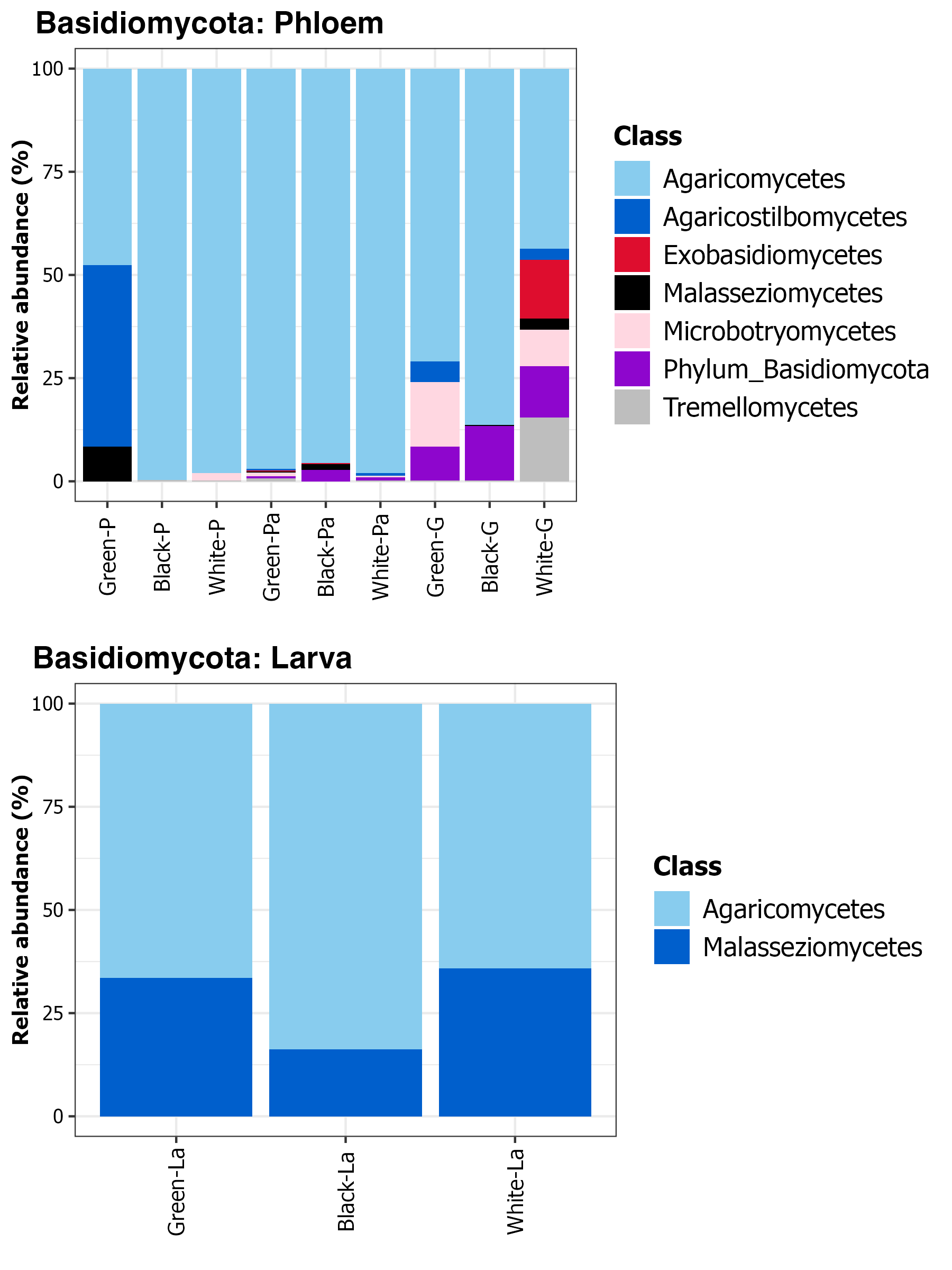


**Fig. S3.** Distribution of the Ascomycetes Classes across all the sample types


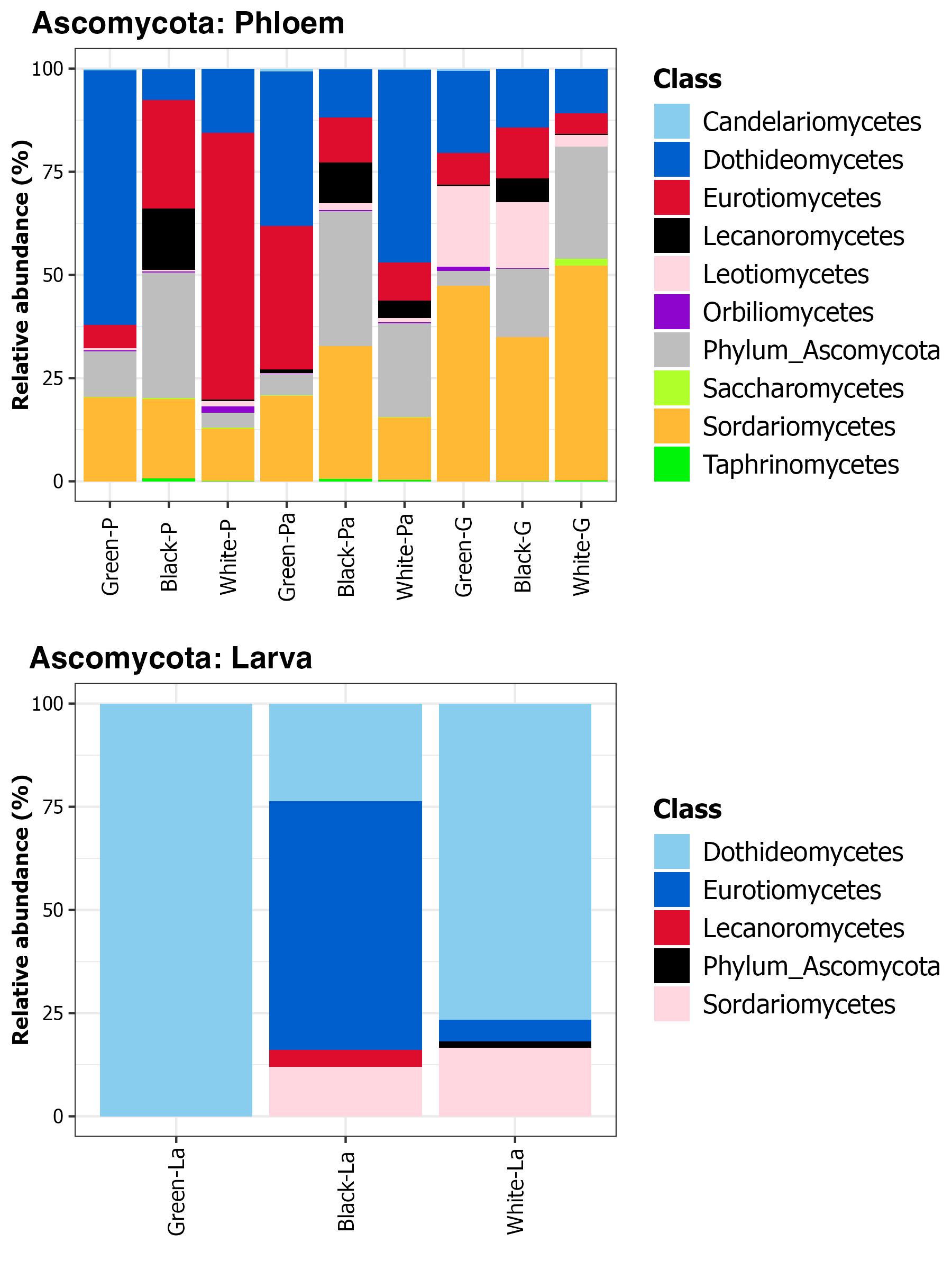


**Fig. S4.** Taxonomic profile of Phyla and Class across all the sample types (bacterial community)


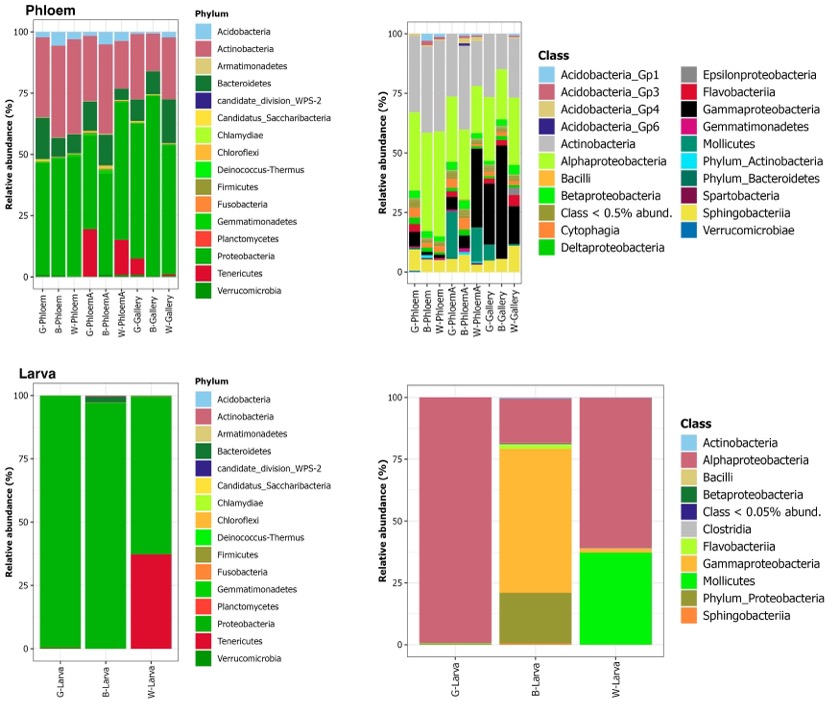


**
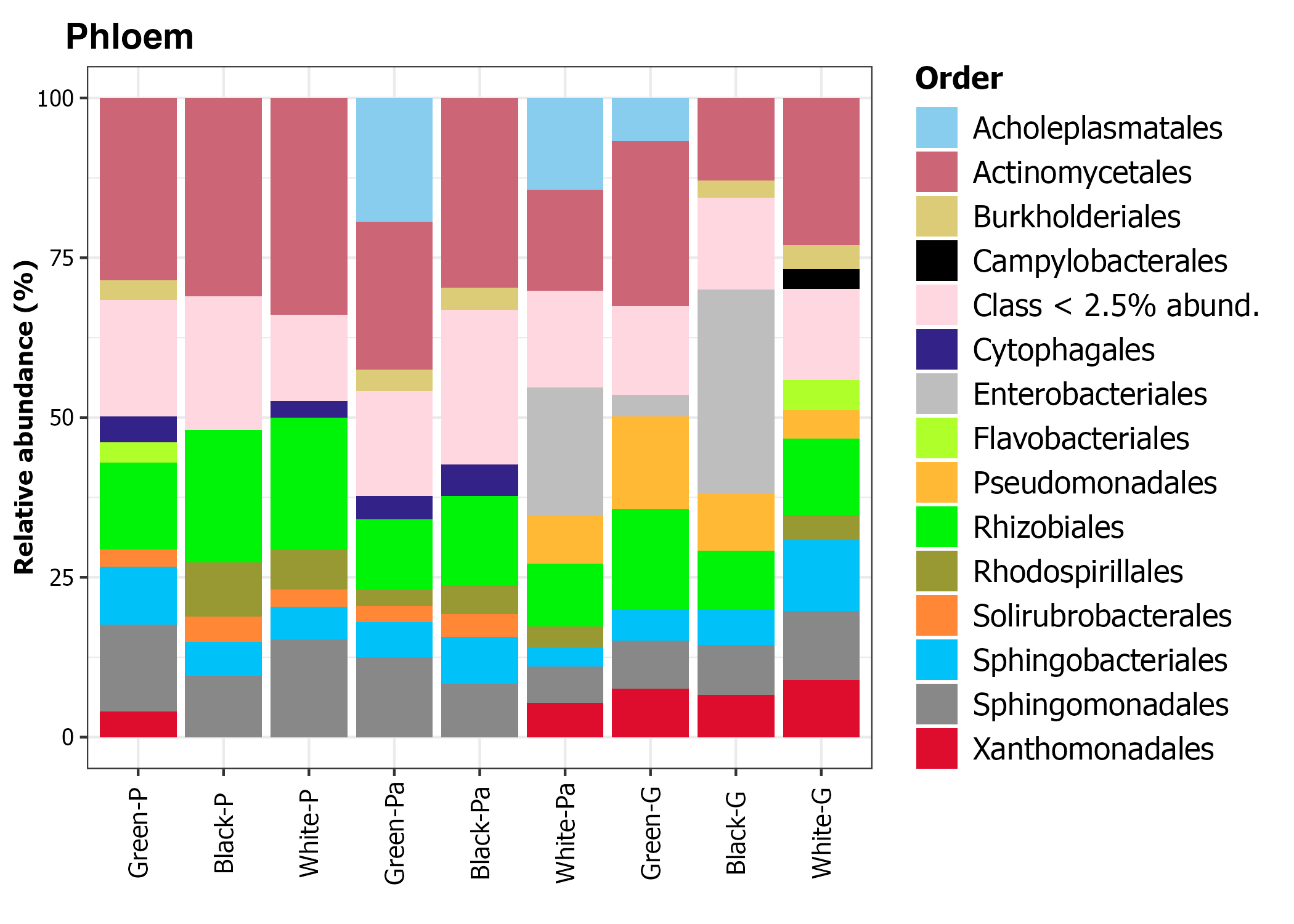
**

**Fig. S5.** Pie chart showing an overview of all the metabolites found in the 24 samples

**
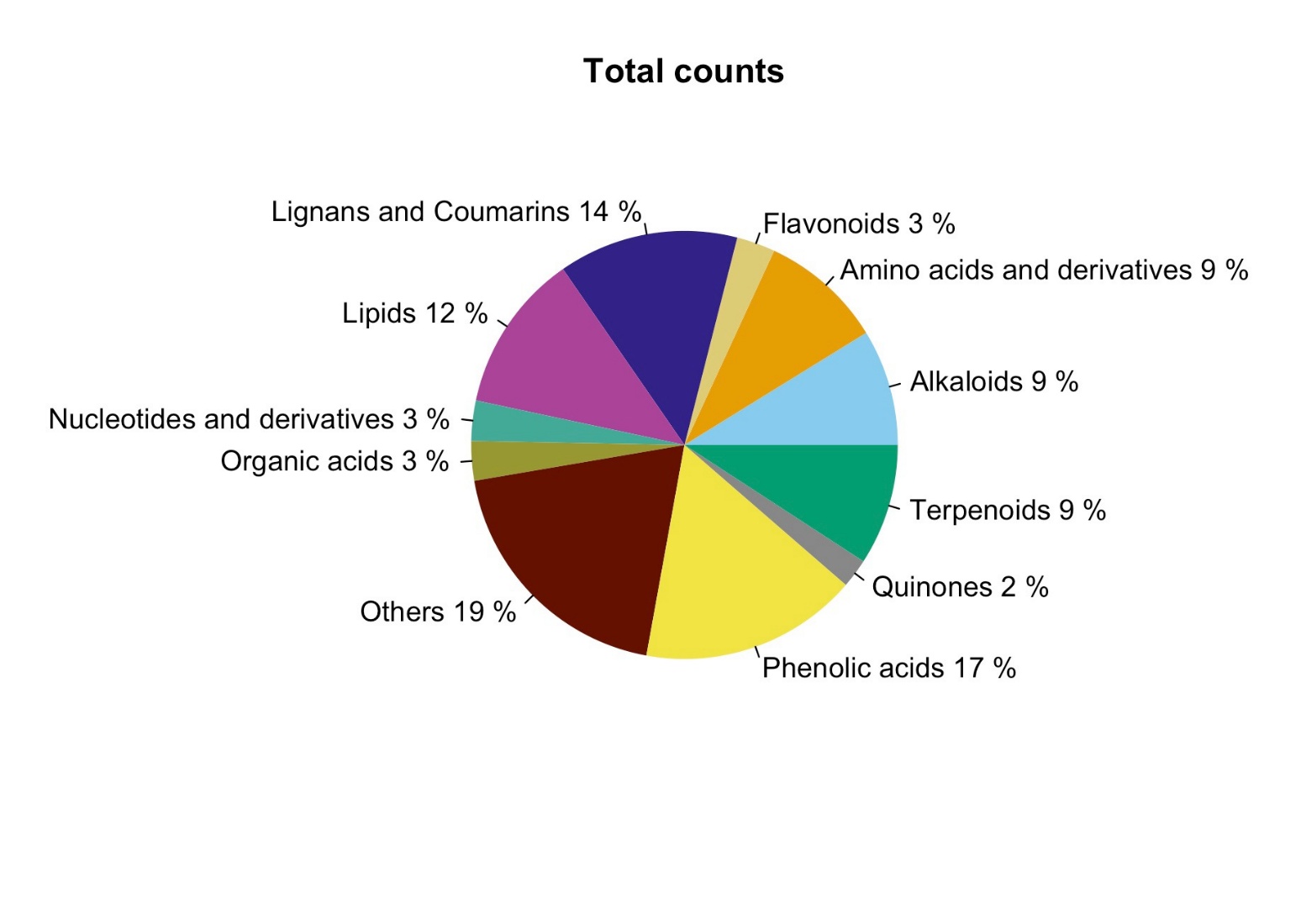
**

**Fig. S6.** Distribution across the ash species for the compounds only detected in one sample type (PhloemA/Gallery). The plain rectangles indicate metabolites not detected in all the PhloemA samples and the dashed rectangles indicate metabolites not detected in all the Gallery samples

**Fig. S7.** Comparisons in the constitutive compounds in the phloem samples (PhloemA). A-OPLS analysis, B- Heatmap performed with the compounds significantly different across the three species

**Fig. S8.** OPLS results of differential analysis that was performed for each ash species between the PhloemA as control compared to the Gallery

**Fig. S9.** Comparison of compounds constitutive in phloem (comparisons between PhloemA sample types) that have been previously implicated in resistance to EAB


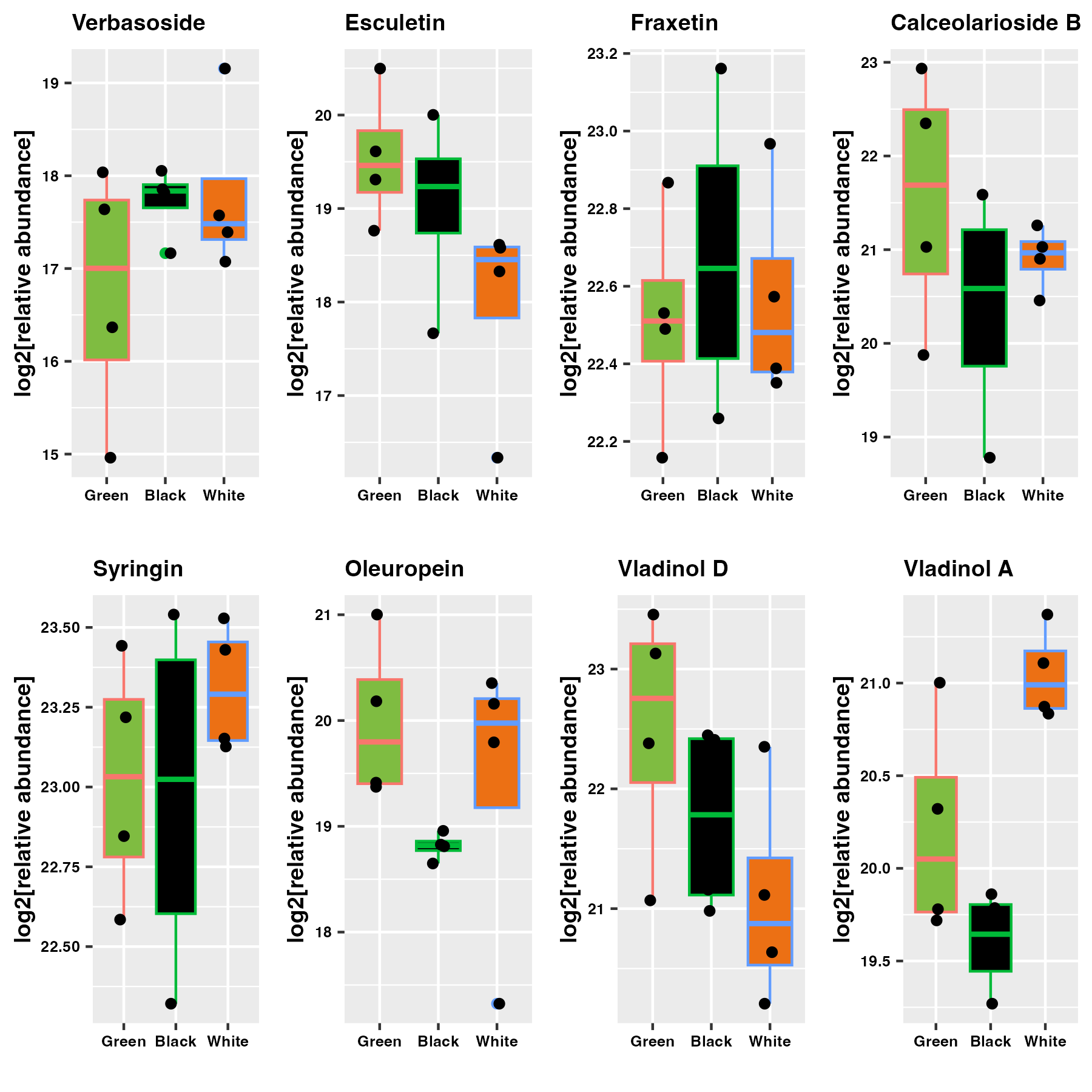


**Fig. S10.** Top twenty most significantly up (red) or down (green) regulated metabolites in each species in OLPS analysis comparing PhoemA to Gallery samples.

**Green ash Black ash**


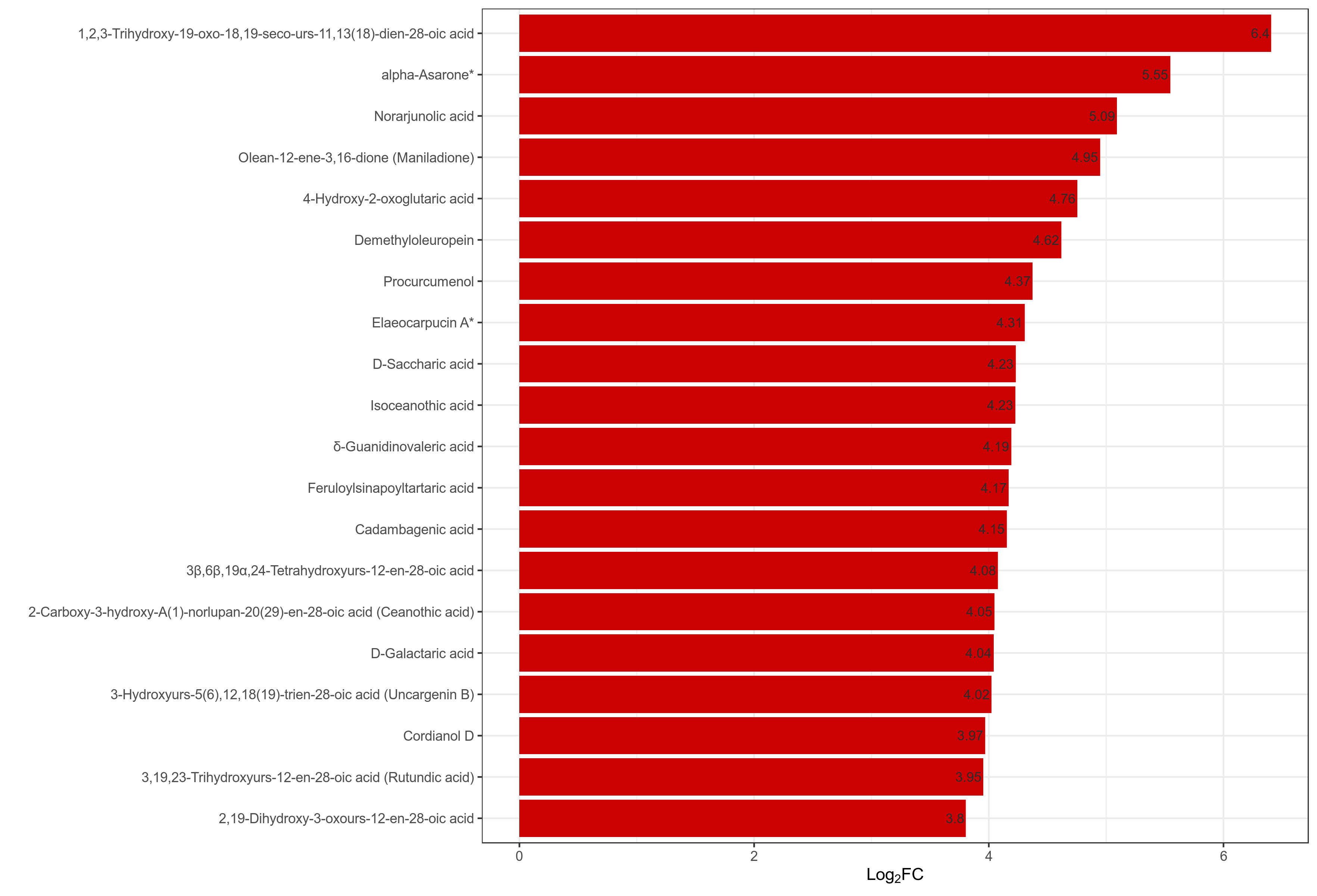

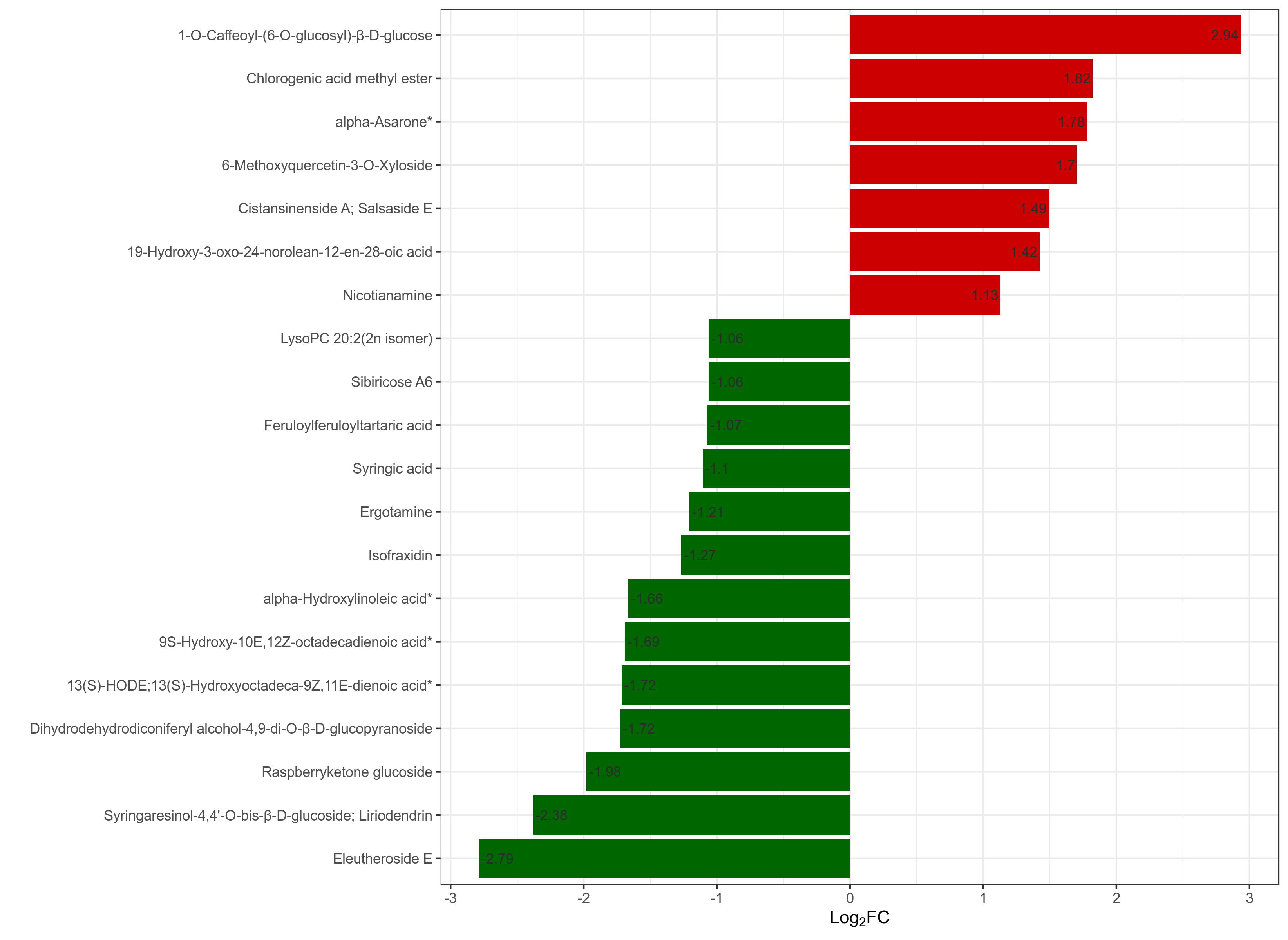


**White ash**


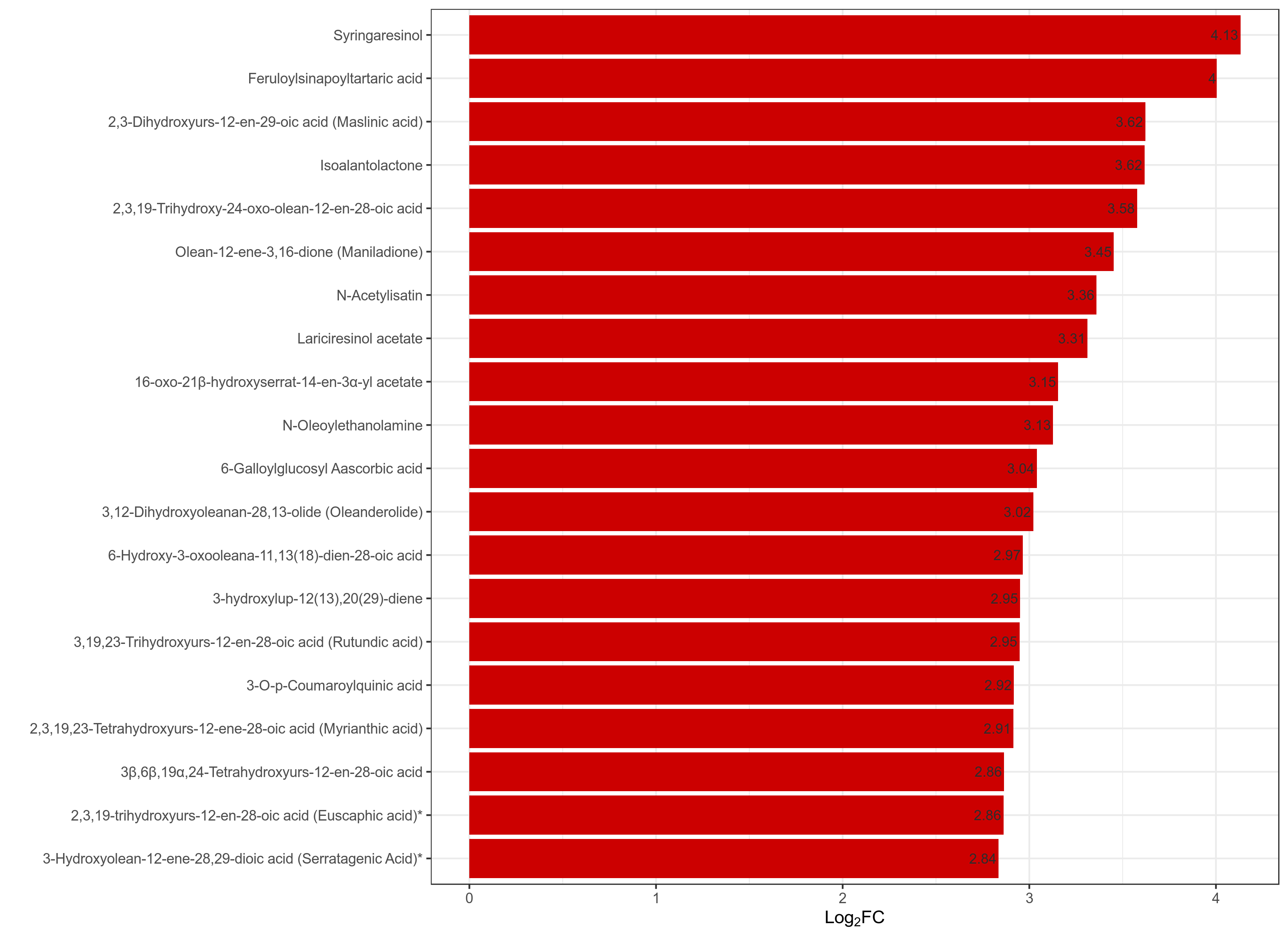

Supplement: Supplementary file 2 — Supplementary Material 2. [file 40793_2026_884_MOESM2_ESM.docx]
